# Supplementary material for: Subjective optimality in finite sequential decision-making
Source: PLoS Comput Biol. 2021 Dec 16;17(12):e1009633. doi: 10.1371/journal.pcbi.1009633 (PMC8675647; doi:10.1371/journal.pcbi.1009633)
Supplement: S1 File — A .docx file containing supporting text and figures. (DOCX) [file pcbi.1009633.s001.docx]

Supporting Information for

**Subjective optimality in finite sequential decision-making**

Yeonju Sin,^†^ HeeYoung Seon, ^†^ Yun Kyoung Shin, Oh-Sang Kwon, Dongil Chung

^†^These authors contributed equally

Correspondence to: Oh-Sang Kwon or Dongil Chung

Email: O-SK ([oskwon@unist.ac.kr](mailto:oskwon@unist.ac.kr)), DC ([dchung@unist.ac.kr](mailto:dchung@unist.ac.kr))

**This PDF file includes:**

Text A

Figs A-J

Supplementary References 1-4

**Text A**

Subjective utility function model justification

Based on the framework of Prospect theory [1], our suggested computational model introduced a subjective value function that includes a reference point and a nonlinear value sensitivity. Note that in our suggested model, we assumed that probability information is not distorted (linear probability weighting function) and that individuals are equally sensitive to gains and losses (loss neutrality). By doing so, we sought for simplicity while examining the impact of given contexts with a reference point, the key premise of Prospect theory [2]. Here, we tested two other models those belong to the same model family to further explore the impacts of simplified elements.

**Expected utility model.** As one of the special cases of the Subjective optimality model, we examined the effect of a non-linear utility function without a reference point. Across all experiments, the Expected utility model (labeled as ExpUtil) showed inferior model fit (ExpUtil: 15139) compared to the Subjective optimality model with a waiting cost (SubjOpt: 15077). In each experiment, on the other hand, these model family (i.e., PT and ExpUtil) showed comparable model fits, equally better than the Optimal decision model (**Fig A in S1 File**). These results suggest that inclusion of a non-linear utility function has an important role in explaining the empirical data.

Further assessment of the estimated parameters included in the ExpUtil revealed rather subtle but crucial lack of plausibility. In the ExpUtil model, mean of the value sensitivity (often referred to as ‘risk preference’) was 2.88±1.04, indicating extremely strong *risk seeking* in individuals. This result is inconsistent with previous reports where typical healthy individuals are reported to have *risk aversion* (smaller than 1) in a gain frame (decision about positive rewards).

**Linear loss aversion model.** Another main component suggested in Prospect Theory other than a reference point is the enhanced sensitivity to losses relative to gains. To directly examine the impact of this ‘loss aversion’, we tested a linear utility function with inclusion of a reference point and a loss aversion. Across all experiments, the linear loss aversion (labeled as LinearLA) model showed inferior model fit (LinearLA: 15288) compared to the Subjective optimality model with a waiting cost (SubjOpt: 15077). In each experiment, on the other hand, these model family (i.e., PT and LinearLA) showed comparable model fits, equally better than the Optimal decision model (**Fig A in S1 File**).

Further assessment of the estimated parameters included in the LinearLA revealed rather subtle but crucial lack of plausibility. In the LinearLA model, mean of the loss aversion parameter was 0.23±0.03 (reference point = 98.77±15.63), indicating extremely strong *loss* *seeking* in individuals. This result is inconsistent with the rich previous literature on *loss* *aversion* that typical healthy individuals are reported to show (larger than 1).

**Discussion about non-linear utility function family.** Based on the implausible parameters estimated for the ExpUtil and LinearLA models, we can conclude that it is unlikely for participants to use these values functions in their valuation and decision-making processes. Still, it seems worthwhile to discuss how come the models had comparable model-fit (besides the fact that the Subjective optimality model has one additional parameter (for the ExpUtil model), so that the model was penalized further in model comparison). The atypical parameters in the two alternative models (i.e., LinearLA, ExpUtil) correspond to a convex utility function across the full range of stimuli values. Note that the shape of these value functions provide similar coverage with that of our suggested model, which includes a high reference point (here, 110.51; see **Fig 2B**) in combination with a concave value function for the gains (value above the reference point) and a convex value function for the losses (value below the reference point). This similarity between the shapes of value functions suggest that a broader range of stimuli values would be necessary to better dissociate each model’s explanatory power. Nevertheless, inclusion of the reference point in the Subjective optimality model provided the model a superior generalization capacity across different contexts (e.g., different prior knowledge about maximum, different number of opportunities). We believe such a strength exceeds the aforementioned limitation.

**Fig A. Model comparisons using Akaike Information Criterion (AIC)**

To examine model fit of our suggested computational models, we computed AIC scores of each model at individual-level (smaller AIC score indicates better model fit), and compared AIC score of the Subjective optimality model with a waiting cost (SubjOpt+wc) against other eight alternative models: Subjective optimality model without a waiting cost (SubjOpt), Independent threshold model (IndThresh), Linear threshold model (Linear) [3], Constant threshold model (Const), Optimal model with a waiting cost (Opt+wc), Optimal model (Opt), Linear utility model with loss aversion (LinearLA), and Expected utility model (ExpUtil). Positive ∆AIC scores indicate that the SubjOpt+wc model showed better model fit compared with the alternative model, and vice versa. The red asterisk indicates that 95% confidence interval of $\Delta$AICs does not include 0 in negative direction (i.e., SubjOpt+wc model showed worse model fit). The blue asterisk indicates that 95% confidence interval of $\Delta$AICs does not include 0 in positive direction (i.e., SubjOpt+wc model showed better model fit). **(A-D)** In all experiments, model fits of Const, Opt+wc, and Opt models were considerably worse than that of SubjOpt+wc model. Note that a simple addition of the waiting cost does not improve the explanatory power of the Optimal decision threshold model, suggesting that the shape of subjective utility function has more crucial role. **(A)** In Experiment 1, the SubjOpt and ExpUtil models showed better model fit compared with the SubjOpt+wc model. **(B)** In Experiment 2, the Linear model showed better model fit compared with the SubjOpt+wc model. **(C)** In Experiment 2 (K=10), the SubjOpt+wc model performed significantly better than the IndThresh model, which is due to that the number of parameters in the IndThresh model increases as the number of opportunities increases. **(D)** In Experiment 3, SubjOpt+wc showed better fit than the SubjOpt model indicating that the forced waiting time in the experiment was costly. **(A-D)** Considering the AICs for all experiments and participants together, the best model for the entire data set was the SubjOpt+wc model (AIC_SubjOpt+wc_ = 15077, AIC_SubjOpt_ = 15159, AIC_IndThresh_ = 15160, AIC_Linear_ = 15116, AIC_Const_ = 16759, AIC_Opt+wc_ = 16864, AIC_Opt_ = 21601, AIC_LinearLA_ = 15288, AIC_ExpUtil_ = 15139).

**Fig B. Response times in Experiment 2**

Response times (RTs) for each opportunity in Experiment 2 (K = 5) were (Left) computed against the presented stimuli values. (Right) Regardless of the opportunity, RTs showed negative association with the absolute distance between the presented stimuli and the corresponding decision threshold. That is, participants showed the shortest RTs for the numbers that are farthest from decision thresholds, and vice versa. Error bars represent s.e.m.

**Fig C. Model-based prediction using K=10 data**

Model-based predictions of the decision threshold (blue) made for K=2 and 5 using the K=10 data as a reference showed comparable results with the prediction for K=2 and 10 using the K=5 data as a reference (**Fig 3B**). Note that the confidence interval of the prediction (shaded area) is wider for the prediction based on K=10 data due to the wider variability in the original empirical data (red error bar for K=10). Error bars represent 95% confidence interval.


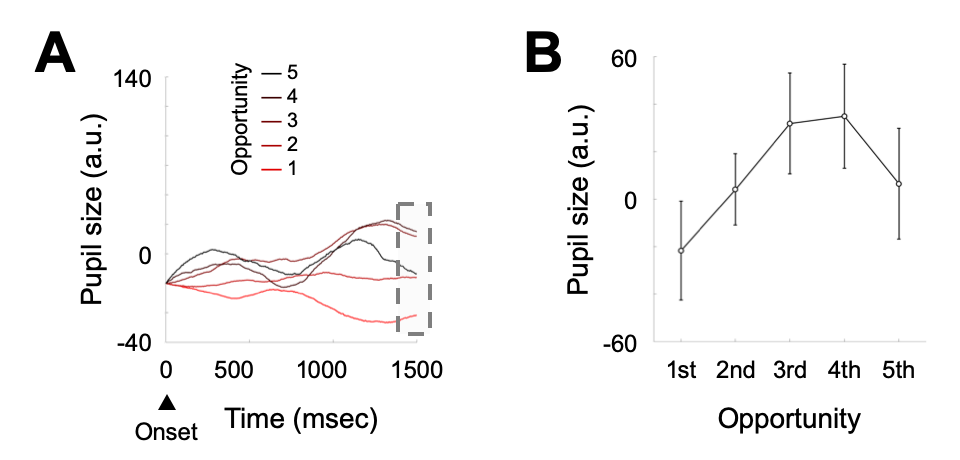


**Fig D. Pupil responses reflect individuals’ arousal level**

**(A)** Pupil size change from the stimuli onset was measured, separately for each opportunity (1^st^, 2^nd^, …, and 5^th^). The pupil sizes at different opportunity were distinctively separable from around 1 sec after the stimuli onset. **(B)** Particularly, pupil sizes at 1500msec after the stimuli onset showed increasing pattern along the stages of opportunities, such that pupil size at the 1^st^ opportunity was significantly smaller compared with that at the 3^rd^ and 4^th^ opportunities (1st vs 3rd: t(17) = 3.05, Cohen’s d = 0.72, p = 0.007; 1st vs 4th: t(17) = 3.07, Cohen’s d = 0.72, p = 0.007). Such linearly increasing pattern in pupil responses along the repeated opportunities suggests that individuals entered higher arousal states as the number of remaining opportunities decreased. At the 5th opportunity, as the last opportunity in K = 5 condition, individuals had to accept any stimuli, which may explain why the pupil size at the 5th opportunity deviates from the linear pattern. Error bars represent s.e.m.

**Fig E. Pupillometry responses from a subset of opportunities**

**(A)** The peak pupil sizes are depicted as a function of the signed distance between stimuli value and the corresponding decision threshold (duplicated from **Fig 5B**). Given the nature of the last opportunity that individuals have no choice but to accept any stimuli values, pupil responses from **(B)** opportunities 1 to 4, **(C)** opportunity 1 separately from opportunities 2 to 4, and **(D)** opportunity 5 alone are illustrated in separate panels. Shades represent s.e.m.

**Fig F. Pupillometry responses with an inclusive selection criterion**

We repeated the pupil data analyses with a more inclusive trial selection criterion by only excluding those trials that required substantially large proportion (>90%) to be interpolated. **(A)** Pupil size change from the stimuli onset was measured, separately for the accepted (green) and rejected (red) opportunities. Paired comparison between the cases revealed significant pupil dilation for the accepted stimuli (t(17) > 2.11, all *ps* < 0.05) at the early stage after the onset (570ms to 700ms), and again at the later time (1184ms to 1500ms). Only the latter cluster remained significant after correcting for multiple comparisons using a cluster-based permutation method [4] (*p_corrected_* = 1.50e-4). **(B)** The peak pupil size between the onset of the stimulus and 1500 msec after the onset was depicted as a function of the signed distance between stimuli value and the corresponding decision threshold. Pupil size decreased as a function of the absolute distance between the decision threshold and value of the presented stimuli (slope = -0.0103, t(17) = -3.27, Cohen’s d = -0.77, *p* = 0.0045). **(C)** Individuals who had higher value sensitivity in their estimated parameter (median split; red) showed relatively high pupil dilation compared to individuals who had low value sensitivity (blue). This positive correlation between value sensitivity and pupil dilation was statistically within the signed decision difficulty (Value – Threshold _i-th_) ranging from -17 to 40 (Pearson’s correlation *r* > 0.47, all *p*s < 0.05). The significance remained valid after correcting for multiple comparisons using a cluster-based permutation method [4] (*p*_corrected_ = 0.012). Shades represent s.e.m.

**Fig G. Best fitting parameters**

The Subjective optimality model with a waiting cost was used to estimate the four parameters best explaining individuals’ behavioral choices. **(A)** The estimated nonlinear value sensitivity (⍴) was comparable among all four separate experiments (Experiments 1, 2 (K = 5), 2 (K = 10), and 3: F(3, 78) = 0.27, *p* = 0.84). **(B)** We hypothesized that the reference point in Experiment 2 (K=10) would be higher than that in Experiment 2 (K=5), because the expected future earnings was higher. Indeed, the reference point for Experiment 2 (K=10) was higher, and the difference was statistically significant (one tailed t-test: t(39) = -1.85, Cohen’s d = -0.58, *p* = 0.036). Comparing the reference points among all four experiments showed marginal difference (F(3, 78) = 2.71, *p* = 0.0504). **(C)** There was a significant difference in decision variability between experiments (F(3, 78) = 7.13, *p* = 2.69e-4). Post-hoc tests revealed that the difference originates from the higher decision variability in Experiment 1 (Tukey test: Exp. 1 vs. 2 (K=5): *p* = 0.048; Exp. 1 vs. 2 (K=10): *p* = 0.0007; Exp 1 vs. 3: *p* = 0.0008). **(D)** Waiting costs were larger than zero in Experiment 2 (K=10) and Experiment 3 (Experiment 1: t(19) = -1.84, Cohen’s d = -0.41, *p* = 0.082; Experiment 2 (K=5): t(20) = 0.37, Cohen’s d = 0.08, *p* = 0.71; Experiment 2 (K=10): t(19) = 4.36, Cohen’s d = 0.97, *p* = 3.36e-4; Experiment 3: t(20) = 63.51, Cohen’s d = 13.86, *p* = 1.51e-24). There was a significant difference in waiting costs between experiments (F(3, 78) = 28.29, *p* = 1.75e-12). Error bars indicate s.e.m.

**Fig H. Decision thresholds along the course of the task rounds**

Potential effects of learning on decision behaviors were examined by separately estimating the thresholds in the first half (round 1 to round 100) and the second half (round 101 to round 200) of each experiment. We applied repeated measures ANOVA with the opportunity (1st, to K-1st) and the round (first half vs second half) as fixed factors, and participants as a random factor. In Experiment 1, the decision thresholds decreased from the first half to the second half and the change was statistically significant (F(1,19) = 14.4, *p* = 0.0012). However, the main effect of round was not statistically significant in Experiments 2 and 3. In Experiment 1, participants might have initially set the thresholds relatively high, and took the first half of the task adjusting their thresholds. This behavioral pattern in Experiment 1 is likely due to the fact that participants were not informed of the maximum value unlike the other experiments. The interaction effect between opportunity and round was not statistically significant (F(3,57) = 2.61, *p* = 0.060). In Experiment 2 (K=5) and Experiment 2 (K=10), neither the main effect of round nor the interaction effect was statistically significant. In Experiment 3, the interaction effect between the round and opportunity was statistically significant (F(3,60) = 3.35, *p* = 0.025). Note that the threshold at 4^th^ opportunity in Experiment 3 tended to increase away from the optimal threshold value. This pattern suggests that the learning across rounds does not always move toward the optimal strategy but moves toward the thresholds that reflect the subjective value.

Fig I. Effects of past stimuli values on the subsequent choice about the current value

Effects of past stimuli values on the current decision were examined by applying the logistic regression analyses using aggregated data set from Experiment 1, Experiment 2 (K=5), and Experiment 3. Results show that the probability of accepting the current value increases as the current value increases and as the past value decreases. Each panel depicts regression coefficients from a logistic regression using choice data at an indicated opportunity. Blue asterisks indicate statistically significant weights in positive direction (*p* < 0.05), and red asterisks indicate statistically significant weights in negative direction. It is important to note the considerable differences between betas for the current stimuli and those for the past stimuli; although some of the betas for the past stimuli values were statistically significant, the size of betas were minimal compared to the weight on the current value.

Fig J. Number of accepted occasions at each opportunity

The number of accepted occasions is plotted for each opportunity in each of the four experiments. In Experiment 3, participants accepted the stimuli given at the first opportunity relatively more frequently, which might be the consequence of the enforced waiting time.

Supplementary References

1. Kahneman D, Tversky A. Prospect theory: An analysis of decision under risk. Econometrica: Journal of the Econometric Society. 1979:263-91.

2. Bendor J. Bounded rationality. 2001.

3. Baumann C, Singmann H, Gershman SJ, von Helversen B. A linear threshold model for optimal stopping behavior. Proceedings of the National Academy of Sciences. 2020;117(23):12750-5.

4. Maris E, Oostenveld R. Nonparametric statistical testing of EEG-and MEG-data. Journal of neuroscience methods. 2007;164(1):177-90.
