# Supplementary material for: Subjective optimality in finite sequential decision-making
Source: PLoS Comput Biol. 2021 Dec 16;17(12):e1009633. doi: 10.1371/journal.pcbi.1009633 (PMC8675647; doi:10.1371/journal.pcbi.1009633)
Supplement: Attachment — Submitted filename: Response_to_reviewers_R1.docx [file pcbi.1009633.s002.docx]

Dear Dr. Gershman and Dr. Haith,

Thank you very much for considering our manuscript PCOMPBIOL-D-20-01425 (“Subjective optimality in finite sequential decision-making”) for publication in PLOS Compuational Biology. We appreciate the opportunity to revise and resubmit our manuscript and believe that it has substantially improved with the help of your feedback.

We note that this revision includes an additional 20 behavioral assessed participants that helped confirm the generalizability of our suggested computational model. All the results were consistent with the original conclusion.

We appreciate the reviewers’ interest and positive assessments of our work, including their comments noting that: “Overall, I think that this is an interesting topic that gets a lot of attention in economics and business journals, but could benefit from a neuroscience perspective” (**Reviewer 1**), “the empirical result is a novel contribution to the literature” (**Reviewer 2**), “I commend the authors for pursuing this timely and important question” (**Reviewer 3**).

We also appreciate the constructive criticisms provided by the editor and reviewers and provide below a detailed response to each point with

i) additional analyses,
ii) new data,
iii) more thorough literature review, and
iv) accompanying text.

The reviewers’ comments are fully quoted below in bold; our responses follow each comment. Changes to the submission in response to the  reviewers’ comments are highlighted in blue in the revised manuscript and supplementary materials and also reproduced or summarized below.

--

**Dear Dr Chung,**

**Thank you very much for submitting your manuscript "Subjective optimality in finite sequential decision-making" for consideration at PLOS Computational Biology.**

**As with all papers reviewed by the journal, your manuscript was reviewed by members of the editorial board and by several independent reviewers. The reviewers raised a significant number of concerns about the paper. Some of these concerns relate to the modeling framework, with reviewers suggesting the paper does not go far enough in considering alternative models and that the appeal to prospect theory might be a more complex model than can be justified. The reviewers also noted shortcomings in the discussion of relevant prior literature, as well as concerns about aspects of the data analysis, including possible selection biases and learning-related effects. Finally, all reviewers noted that the pupil dilation results are somewhat disconnected from the main questions that the paper considers, at least in the way that the paper is currently framed.**

**Although a substantial number of concerns were raised, we would like to invite the resubmission of a significantly-revised version that takes into account the reviewers' comments.**

**We cannot make any decision about publication until we have seen the revised manuscript and your response to the reviewers' comments. Your revised manuscript is also likely to be sent to reviewers for further evaluation.**

**When you are ready to resubmit, please upload the following:**

**[1] A letter containing a detailed list of your responses to the review comments and a description of the changes you have made in the manuscript. Please note while forming your response, if your article is accepted, you may have the opportunity to make the peer review history publicly available. The record will include editor decision letters (with reviews) and your responses to reviewer comments. If eligible, we will contact you to opt in or out.**

**[2] Two versions of the revised manuscript: one with either highlights or tracked changes denoting where the text has been changed; the other a clean version (uploaded as the manuscript file).**

**Important additional instructions are given below your reviewer comments.**

**Please prepare and submit your revised manuscript within 60 days. If you anticipate any delay, please let us know the expected resubmission date by replying to this email. Please note that revised manuscripts received after the 60-day due date may require evaluation and peer review similar to newly submitted manuscripts.**

**Thank you again for your submission. We hope that our editorial process has been constructive so far, and we welcome your feedback at any time. Please don't hesitate to contact us if you have any questions or comments.**

**Sincerely,**

**Adrian M Haith**

**Associate Editor**

**PLOS Computational Biology**

**Samuel Gershman**

**Deputy Editor**

**PLOS Computational Biology**

***************************

**Reviewer #1: This is an interesting paper looking at decision making in sequential search problems, a.k.a. secretary problems. The authors hypothesize that rather than using an optimal value maximizing strategy, participants instead have a reference point and behave as if they are maximizing utility with respect to that reference point. They test one prediction of this in Experiment 2, by showing that people appear to have higher reference points, and thus thresholds, when there are more opportunities. In Experiment 3 they also collect pupil dilation data and study its relation to choice outcomes and difficulty. They show that pupil dilation increases with difficulty, but is also higher when accepting opportunities.**

**Overall, I think that this is an interesting topic that gets a lot of attention in economics and business journals, but could benefit from a neuroscience perspective. I also think the authors are likely onto something with the Prospect Theory story (but see below). My enthusiasm is dampened by a few issues, some which could be addressed by expanding the scope of the literature review, others which would require new analyses and could change the takeaway messages of the paper. My view is that there are a collection of (potentially) interesting results here, but they don’t all hang together very well, and they don’t quite tell a compelling, coherent story.**

**One potential major problem that I see is an issue of selection bias in the analyses. There will always be some subject-level heterogeneity in behavior, in this case in the thresholds that participants use. Subjects with lower threshold strategies will tend to accept earlier opportunities and so be underrepresented at subsequent opportunities. This will make it look like the thresholds are relatively flat across opportunities, compared to the reality. This is particularly an issue for Experiment 2, where the authors compare behavior in Opportunity 1 (out of 2) to Opportunity 4 (out of 5). The authors find that participants in the former case have a lower threshold than those in the latter, which seems like an important finding, and provides crucial support to their Prospect Theory explanation. However, it could simply be that all the “low threshold” participants have disappeared by Opportunity 4, while the entire population is present in the Opportunity 1 analysis. The authors could try to address this by including trials where participants accepted prior to Opportunity 4, and estimating what their threshold would have been in Opportunity 4, based on what they accepted earlier. This would seem to require some extrapolation, so I’m not sure this analysis would ever be clear cut. The authors might want to look at the Heckman correction for missing observations.**

Thank you for the comment. As the reviewer pointed out, due to the nature of the sequential decision-making paradigm, it is inevitable that there is less data that could be used to inform participants’ decision thresholds at the later opportunities compared to the earlier opportunities. However, we would like to point out that the threshold estimation was conducted at *individual level* at each opportunity, and also that all individuals did have enough number of rounds (23-123 (mean=71.3±5.7) out of 200 rounds in total) where they held up their choices until the fourth opportunity in Experiment 2. Therefore, it must not be the case that the “low threshold” participants have disappeared at the later opportunity inducing selection bias. To clarify that the empirical decision thresholds were estimated at individual level, we edited the section as follows.

[Page 8, Line 152] To examine whether individuals follow such decision processes, we calculated empirical thresholds—the value where individuals were equally likely to accept or reject—from 20 participants’ behavioral choices (male/female = 10/10, age = 22.85 ± 1.31 years) (**Fig. 1B**).

🡪 To examine whether individuals follow such decision processes, we calculated empirical thresholds—the value where individuals were equally likely to accept or reject—at individual level using each individuals’ behavioral choices (male/female = 10/10, age = 22.85 ± 1.31 years) (**Fig. 1B**).

**The previous point notwithstanding, I have to question the evidence for the Prospect Theory (PT) explanation. While PT is well established and offers a sensible explanation here, I wonder what it adds empirically? How does this model with a reference point do more than just fitting a threshold model where the initial threshold and threshold decay rate (or time cost) vary across participants? Where is the model comparison with Optimal+C for instance? I guess I’m wondering whether the PT model yields predictions that can be falsified or whether it is so flexible that it could match just about any pattern of results? This is a crucial part of the paper, and I feel like the evidence for it is lacking.**

We agree that empirical usefulness is very important. To address the reviewer’s question, we added three additional models and conducted a formal model comparison using Akaike Information Criterion (AIC) examining the model fit of all seven potential models: i) Constant threshold model, ii) Optimal model, iii) Optimal model with a waiting cost, iv) Independent threshold model (the threshold for an opportunity is estimated independently), v) Linear threshold model (the threshold changes linearly across opportunities) (Baumann et al., 2020), vi) Subjective optimality model without a waiting cost, and vii) Subjective optimality model with a waiting cost. Among the seven models, the model that best explains empirical human choice data (lowest AIC score) across all experimental data sets was the Subjective optimality model with a waiting cost. The following figure now is added as figure S1 in Supporting Information (SI).

**Revision figure 1 (now added as figure S1 in SI).** Model comparisons using Akaike Information Criterion (AIC)

More importantly, as the reviewer pointed out, the Subjective optimality models (with and without a waiting cost) offers a sensible explanation and, as a result, can provide critical predictions unlike those models that only offer descriptive functional form. Here are two examples. First, the value sensitivity in the Prospect Theory (PT) has been shown to be less than 1 in various studies (marginally diminishing values in gain domain and marginally increasing values in loss domain). By applying value sensitivity less than 1 to the Subjective optimality models, one can predict that change of decision thresholds across opportunities should be shallower than the threshold changes the Optimal model predicts; this is a prediction that is falsifiable. Second, the model accounts for why the threshold at the opportunity that precedes the last varies depending on the context. Note that following the Optimal decision model, the opportunity that precedes the last always should have the same expected value regardless of the context (total number of opportunities). We showed in Experiment 2 that this model-based quantitative prediction, which is again falsifiable, is confirmed by the experimental data. We cannot rule out the possibility that yet another model that suggests a descriptive functional form proves to be a better model for the data. However, we believe that the model we suggest has strength over the descriptive models in that it provides sensible explanations and predictions that are coherent with the existing knowledge of human decision making.

**The pupil dilation analysis (Experiment 3) is a very important part of the paper, but it feels somewhat tacked on and disconnected from the rest of the story. This is in part due to the fact that the authors never mention pupil dilation in the introduction of the paper. They do briefly discuss it prior to Experiment 3, but their literature review is a single sentence, and their hypotheses are very vague, stating only that “pupil diameter may capture value of stimuli, decision difficulties, and final choices…” What exactly did the authors hope to see here? How do these pupil results contribute to our understanding of pupil dilation, or of the secretary problem?**

We regret the disconnection. It is now broadly accepted that computational modeling approach has its strength providing mechanistic explanation underlying individuals’ decisions. There could be numerous alternative algorithmic models in a given context, but the models get better support when provided with additional sets of evidence showing that information processes in the brain are consistent with the pattern the model suggests. In the current study, pupil responses were used as a neurophysiological confirmatory measure of our suggested computational model. To clarify our approach, we added the following paragraph in the Introduction section:

[Page 6, Line 109] In conjunction with the computational modeling approach, we recorded individuals’ pupil dilation responses and analyzed their association with participants’ choice behaviors to corroborate our suggested model. A rich set of evidence suggests that pupil dilation (or contraction)—a neurophysiological signature coinciding with norepinephrine levels in the brain—reflects not only individuals’ arousal level [1, 2], but also cognitively complex information, such as value [3, 4], uncertainty [1], surprise [5], cognitive conflict [6, 7], and choice [8]. More specifically, recent studies showed that pupil-linked neuromodulatory systems have a critical role in decision formation, rather than simply in reflection of the decision consequences [8], and moreover that the size of pupil responses is associated with individuals’ choice patterns [9, 10].

Based on these previous studies, we hypothesized that individuals’ pupil responses would be linked to their decision processes in three folds; i) pupil responses may be larger to the options that will be accepted versus rejected, ii) pupil dilation may reflect the deviation between option values and decision thresholds, supporting value- and threshold- based decision processes, and iii) pupil responses in individuals who show larger value sensitivity may be larger. To test these hypotheses, we conducted an additional experiment (Experiment 3) where actions (choosing to accept or reject) were temporally dissociated from physiological responses to stimuli; participants (N = 24, male/female = 12/12, age = 22.67 ± 2.28 years) were not allowed to make a choice until an audio cue was played. All the other settings were equal to Experiment 2 where participants had up to five chances (K = 5). Non-overlapping samples were obtained from each experiment (see **Methods** for detailed procedures).

[Page 14, Line 290] As noted earlier, we drew upon previous findings suggesting the role of pupil-linked neuromodulatory systems in decision formation [8] and the association between pupil responses with individuals’ choice patterns [9, 10], and hypothesized that participants’ pupil responses would reflect their subjective valuation defined within our suggested computational model: i) deviation between option values and decision thresholds, ii) acceptance versus rejection decisions, and iii) individuals’ value sensitivity.

**Finally, on a related point, I found the connections to the literature somewhat lacking. There has been a lot of work on pupil dilation by people like Jonathan Cohen, Yale Niv, Robert Wilson, Kerstin Preuschoff, Eran Eldar, Joshua Gold, etc. The authors could do a much better job situating their results in that literature. More importantly, there has been quite a lot of work on consumer search problems in economics and business, yet there are only a handful of references here (only 3 in the introduction!). A quick Google Scholar search yields many results for the combination “consumer search prospect theory”, with relevant papers by e.g. Kogut, 1990. Prospect Theory is so pervasive in JDM research that there must be more related papers here.**

We thank the reviewer for suggesting relevant literature. As noted at the earlier comment, the following edits were made to better position our manuscript in relation to previous studies.

[Page 6, Line 109] In conjunction with the computational modeling approach, we recorded individuals’ pupil dilation responses and analyzed their association with participants’ choice behaviors to corroborate our suggested model. A rich set of evidence suggests that pupil dilation (or contraction)—a neurophysiological signature coinciding with norepinephrine levels in the brain—reflects not only individuals’ arousal level [1, 2], but also cognitively complex information, such as value [3, 4], uncertainty [1], surprise [5], cognitive conflict [6, 7], and choice [8]. More specifically, recent studies showed that pupil-linked neuromodulatory systems have a critical role in decision formation, rather than simply in reflection of the decision consequences [8], and moreover that the size of pupil responses is associated with individuals’ choice patterns [9, 10].

We fully acknowledge and agree that Prospect Theory has been used broadly in the field of JDM to explain human decision-making. Following the reviewer’s suggestion, we added the following paragraph citing relevant papers.

[Page 5, Line 84] In various psychological studies, including an optimal stopping problem in the mate choice domain [11] and consumers’ purchasing decisions [12, 13], it was suggested that individuals’ heuristic valuation, the framework of Prospect theory [14], underlies the biases observed in their decision patterns [15]. Specifically, the Prospect theory has shown that individuals’ choices are guided by subjective valuation of potential gains and losses relative to the context where they are situated [14]. We constructed a computational model that adopts this concept of ‘reference point’ and examined whether the process of subjective valuation explains how individuals’ decision processes deviate from the optimal decision strategy. In addition, to capture individual differences in subjective valuation, we hypothesized that nonlinear value sensitivity (i.e., concave value function for gains and convex value function for losses) [14] and waiting cost [16, 17] parameters would take crucial parts in determining individuals’ decision patterns (see **Methods** for detailed model structure).

**Other comments:**

**Ratcliff 1978 is not a great reference for easier decisions being faster. That is a paper about memory retrieval. The authors might consider references on value-based decision making, e.g. Mosteller & Nogee 1951; Clithero 2018; Konovalov & Krajbich 2019.**

Thank you for the suggestion. We reviewed and cited the references the reviewer suggested.

~ require shorter response times (RT) (Ratcliff, 1978)

→ require shorter response times (RT) [18, 19, see 20 for review]

**It might be helpful to display something like violin plots of accepted numbers for each of the opportunities. Is the distribution really Gaussian, or close to it?**

As suggested, we now present a violin plot of accepted numbers as figure S8 in SI. No abnormality in the shapes of distributions was found.

**Revision figure 2 (now added as figure S8 in SI).** Number of accepted occasions at each opportunity.

**Doesn’t the declining threshold result rule out a flat threshold strategy? How does Fig. 1c look if you use the optimal thresholds instead?**

We apologize for the confusion. The x-axis in figure 1c (i.e., Value - Threshold) is not based on the ‘flat threshold strategy’. Instead, we take into account the varying levels of threshold along the opportunities; for example, x-axis for the first opportunity is [Value - Threshold_1st opportunity_] and for the second opportunity is [Value - Threshold_2nd opportunity_]. To clarify, we edited the label to “Value - Threshold _i-th_”.

To address the second part of the reviewer’s question, we replaced the thresholds we used in the original figure (from behavioral choices) with the optimal thresholds.

**Revision figure 3.** Response time for each opportunity as a function of signed distance between the presented stimuli values and the optimal thresholds.

As expected, compared to figure 1c (using empirical threshold rather than optimal threshold), the symmetric inverse V shape became less evident. This again suggests that response time for behavioral choices does not match with the choice difficulty defined based on the optimal threshold model.

**“till” —> until**

We edited the manuscript (Page 10, Line 197) as suggested.

**It seems worth noting that if time is costly, you would expect even lower thresholds than “optimal”, not higher. This is true in Experiment 3, but not Experiment 1 or 2.**

We fully agree with the reviewer’s comment that the waiting cost itself has an impact of lowering thresholds. However, here are two reasons why the threshold patterns were different among the experiments, and why the thresholds were not simply lower than the optimal. First, we would like to emphasize again that a combination of value sensitivity, reference point, and waiting cost is what determines the thresholds, rather than the waiting cost itself. Second, note that Experiment 3 cannot be directly compared with Experiments 1 and 2, because it was tailored to examine pupil dilation; in Experiment 3, we introduced participants a forced waiting time of 3 seconds per opportunity, in contrast to a freedom of immediate response in Experiments 1 and 2. As expected, the estimated waiting costs in Experiments 1 and 2 were indeed not different from zero, while the estimated waiting cost in Experiment 3 was significantly larger than zero. We edited the discussion section to clarify this point (Page 19, Line 400).

**Why compare models with likelihood ratio and not something like AIC/BIC/DIC/WAIC? I also was unclear exactly why the chi-square test has ~20-60 degrees of freedom. I assume this is because each subject has their own parameter value. What if instead you modeled it like a random-effects model, where there is a population-level mean and variance for the parameter? Then there would only be 2 degrees of freedom. On a related note, Table S1 could do a better job indicating which model wins the comparison.**

We used likelihood-ratio tests in the initial submission because the models are in nested relationships with one another. As noted above, we now included three additional models that are not nested (Independent threshold model, Linear threshold model, Optimal model with a waiting cost). Thus, we computed the Akaike Information Criterion (AIC) scores to compare the model fits (now included as **Fig. S1** in SI). Specifically, we computed the AIC scores of each model at individual level and examined the distribution of ∆AIC for a formal model comparison.

To better visualize the model comparison result, we now replaced the original Table S1 with the following figure, added as figure S1 in SI.

**Revision figure 4 (now added as figure S1 in SI).** Model comparisons using Akaike Information Criterion (AIC).

**In the model, I take it that U(theta[i+1]) means the expected utility of continuing the game, not the expected utility of accepting that amount? This could be made clearer.**

We regret the lack of clarity. U (now edited to Util) refers to the subjective value function itself (Page 30, Line 635). *ϑ*[i+1] is the decision threshold at [i+1]th opportunity in the objective value domain, which is the certainty equivalent value of the expected utility of continuing the game (covering the opportunities i+2 to the last). In other words, as the reviewer mentioned, U(*ϑ*[i+1]) indicates the expected utility of continuing the game.

To clarify this, we made edits as follows.

[Page 30, Line 646] ~ where U^-1^(.) indicates an inverse function of the aforementioned subjective value function.

→ ~ where Util^-1^(.) indicates an inverse function of the aforementioned subjective value function and Util(ϑ[i+1]) indicates the expected utility of continuing the game.

**Fig.2B is missing the optimal behavior.**

We edited the figure to include a blue dotted straight line that represents an objective value function (i.e., a value function used for the optimal behavior). Figure 2 is now edited as below:

**Revision figure 5 (now added as figure 2).** Subjective optimality model.

**Did the authors really hypothesize that the wait time would increase the cost of waiting and thus lower thresholds, or was the wait time just something they did for the pupil measurements?**

As described in the manuscript (Page 14, Line 290), the forced waiting time was implemented to temporally dissociate choice from other cognitive processes that are potentially reflected in pupil dilation data. However, when implementing the waiting time, we did hypothesize that the forced waiting time would increase mental cost of continuing to the later opportunities. The waiting cost estimated from our suggested computational model supported this hypothesis, such that the cost was larger in Experiment 3 compared to either Experiments 1 or 2.

**Shouldn’t the reference point decrease if you know you’re going to be more impulsive and choose earlier?**

If one has a metacognitive understanding of her/his impulsivity relative to the optimal (and non-impulsive) decision strategy, one would be able to use the knowledge in adjusting her/his own reference point. It is important to note that if one’s reference point changes, the change would again affect decision thresholds, subsequent choices, and in turn, their impulsivity. As we describe at line 193 (Page 10), we assume that the time it took for individuals to establish their reference points was negligible.

**Is the peak in pupil dilation near threshold simply due to the fact that these decisions are taking longer, and pupil dilation continues to rise with time? To help address this question, it would be helpful to see a version of Fig. 6a (or Fig. S2a) where the curves are broken down into |value-threshold| bins. This continuous rise in pupil dilation over time is well documented in the literature, include here (Fig. 6a). Along those lines, I wonder how Fig. 6B would look if the authors used something like “peak pupil dilation” rather than a specific time like 1500 ms?**

We thank the reviewer for the constructive and critical comment. First, as suggested by the reviewer, we depicted pupil size changes from the onset to the 1500 msec after the stimulus's onset, binned by | Value-Threshold | (**Revision figure 6**). The resulting figure manifests that the pupil size at 1500 msec, which was chosen somewhat arbitrarily, may not be the best indicator of the pupil response to the presented stimulus value. As the reviewer pointed out, the pupil size at 1500 msec can be confounded by continuous rise in pupil dilation.

**Revision figure 6.** Pupil size changes binned by the signed distance between stimulus value and decision threshold (Value – Threshold) from the stimuli onset to 1500 msec.

To avoid this confounding effect, we agree with the reviewer that it is better to use ‘peak pupil dilation’ to measure the pupil responses to the stimulus value. We modified all related analyses and figures using peak pupil dilation. As shown below, the results were qualitatively similar with the original figures.

**Revision figure 7 (now added as figure 5B).** Peak pupil dilation as a function of signed distance between stimulus value and decision threshold (Value – Threshold _i-th_).

**“humans in general have optimistic bias” - I disagree with this interpretation of the results. A simpler explanation is that participants simply don’t know the upper bound, but do know that the lower bound is zero. This will naturally cause an “optimism bias”, without any actual optimism.**

We agree with the reviewer’s comment and edited the sentence as follows.

[Page 20, Line 435] This result suggests that humans, in general, have optimistic bias (25), which may diminish or even become inverted in other contexts (e.g., scarce opportunities, mental costs).

→ This result suggests that an optimistic bias [21], potentially generated due to the uncertainty about upper bound in the current study, may diminish or even become inverted in other contexts (e.g., scarce opportunities, mental costs).

**What was the suspicion about the payment structure that some participants expressed?**

We instructed participants that all accepted numbers would be added to their final payoff at the end of the task. However, three participants--when we paid them according to their choices--mentioned that they were surprised because they did not believe that we would actually pay as much as they accepted. We considered such a distrust would have affected participants’ motivation, and thus excluded from further analyses.

**P.31 bottom - “the change of participants’ expectation about mean earning was calculated comparing expected values between conditions…” What exactly does this mean? Using the optimal thresholds?**

Yes we did mean to describe that we used an objective value function rather than assuming any particular subjective value functions.

To clarify, we edited the sentence as follows:

[Page 32, Line 677] To be agnostic about differential subjective valuation under different contexts, the change of participants’ expectation about mean earning was calculated comparing expected values between conditions.

→ Specifically, we calculated the expected value across all opportunities for each condition (K=2, 5, and 10), and assumed their difference to indicate the extent to which participants changed their expectation about earnings from average number of opportunities (K = 5) to either abundant (K = 10) or scarce (K = 2) opportunities.

**Excluding 28% of trials from the pupil-dilation analysis is a lot. Are the results robust to less extreme exclusions?**

We repeated the same pupillometry data analyses using a more liberal trial selection criterion. By excluding 9% of trials that required a substantially large proportion (>90%)

of interpolation, we confirmed that all statistical test results were essentially the same (now added as figure S4 in SI).

**Revision figure 8 (now added as figure S4 in SI).** Pupillometry responses with an inclusive selection criterion.

**The authors mention some pupil-dilation smoothing. They mention “-90 to 60”. What exactly are these numbers? What are the units?**

We regret the lack of clarity. The range “-90 to 60” was the number calculated by subtracting the threshold at each opportunity from the presented stimulus (i.e., Value - Threshold _i-th_).

[Page 34, Line 742] ~ the threshold centered values from -90 to 60

→ ~ the threshold centered values (i.e., Value – Threshold _i-th_) ranging from -90 to 60

**Reviewer #2: Summary:**

**In this paper, Shin et al. examine human choice behavior in a sequential decision problem that is a variant of the classic “secretary problem.” In the problem examined, “candidates” are drawn from a uniform distribution between 0 and 150, and on each round, the chooser decides whether to accept the current candidate or reject it and move on to the next round. Choosers face a total of 200 problems and are paid proportional to the sum of all candidates selected. Across experiments, whether choosers are told about the distribution or not is varied, as is the number of rounds (either two or five rounds are used). Under the assumption that the chooser knows the full distribution of candidates, the optimal solution can be easily obtained using dynamic programming, and takes the form that the chooser accepts any candidate above a certain decision criterion and that the optimal decision criterion only depends on the number of remaining candidates. The primary empirical result, replicated across three experiments, is that human choosers do not adjust their decision criterion as strongly as the optimal solution, with a resulting bias towards higher or lower criteria in different experiments. The authors account for choice behavior across experiments assuming a dynamic programming-like model in which, rather than actual values, candidate values are evaluated in a prospect theory-like manner with a non-linear subjective function relative to a reference point. Measurements of pupil diameter in a third experiment also show that pupil diameter is influence by choices and choice difficulty.**

**Comments:**

**1. Though the empirical result is a novel contribution to the literature, the modeling is less compelling. The authors assume that participants solve the problem in a dynamic programming-like manner, but they do not do enough to establish this basic assumption. They do not adequately explore alternative possibilities enough to make such a claim. The only alternative model that is considered is a fixed decision criterion model. Several other critical possibilities are not explored, including (a) that participants’ choices were dependent on the candidates they have passed on in the current round and (b) that participants learn over problems how to adjust their decision criterion in order to maximize outcomes. Ruling out these alternatives is particularly important as there are reasons to doubt that human choosers use the kind of backward induction approach proposed here (eg, many examples in game theory where choosers only look a small number of steps forward/backward).**

We thank the reviewer for a very constructive comment. To explore other possible factors that might have affected the decision behaviors more thoroughly, we now i) considered three additional alternative models, ii) quantified the effect of past values on the current decision, and iii) examined the learning effects across rounds.

First, by including three additional alternative models, we compared model fits of seven alternative models: Subjective optimality model without a waiting coast, Subjective optimality model with a waiting cost, Independent threshold model (the threshold for an opportunity is estimated independently), Linear threshold model (the threshold changes linearly across opportunities) (Baumann et al., 2020), Optimal model with a waiting cost, Optimal model, and Constant threshold model. Among the five alternative models and the two subjective optimality models (with and without a waiting cost), the model that best explained participants’ behavioral choices (lowest AIC score) across the entire data sets was the Subjective optimality model with a waiting cost (now added as figure S1 in SI).

**Revision figure 9 (now added as figure S1 in SI).** Model comparisons using Akaike Information Criterion (AIC).

Second, we ran a series of logistic regressions to examine the impact of presented stimulus at the preceding opportunity in subsequent choices. There was a slight hint of negative influence of preceding stimuli on subsequent choices, but the beta coefficients were less than 1/10 of the betas of the current stimulus.

**Revision figure 10 (now added as figure S7 in SI).** Effects of past stimuli values on the subsequent choice about the current value.

Third, we examined potential effects of learning on decision behaviors by estimating the thresholds in the first half (round 1 to round 100) and the second half (round 101 to round 200) of experiments separately. Results showed that the overall thresholds decreased from the first half to the second half in Experiment 1, and the threshold at 4th opportunity moved away from the optimal threshold in Experiment 3. There was virtually no learning effect found in Experiment 2 (K=5) and Experiment 2 (K=10). The decrease of thresholds over the rounds in Experiment 1 is likely due to that the upper limit of the value was not initially informed to the participants in Experiment 1. The fact that the threshold at 4th opportunity in Experiment 3 moved away from the optimal threshold suggests that the adjustment of thresholds across rounds may not solely determined by the process of maximizing outcomes.

**Revision figure 11 (now added as figure S6 in SI).** Decision thresholds along the course of the task rounds.

In all, results showed that the effects of past stimuli values and learning across rounds are statistically significant in some conditions, but the contributions of those factors in shaping the overall decision behaviors are limited. Among those models that potentially can explain the overall thresholds pattern, we believe the Subjective optimal model is the most desirable one in the sense that it best explains empirical data and it provides a sensible account that is coherent with existing knowledge of human decision making.

**2. The motivation for a prospect theory-like evaluation is not entirely clear. Is the reference point meant to be the expected outcome from the problem? If so, why wouldn’t that reference point change over problems as participants learn to better estimate the expected outcomes? And why wouldn’t the reference point change as a function of the remaining number of potential candidates?**

As the reviewer correctly pointed out, in principle one can conceive a model in which the reference point changes over problems as a result of learning and/or a model in which the reference point changes as a function of the remaining opportunities. However, empirical data do not support those models. First, as we described in the response to the comment above, the effect of learning over the rounds is tangential to determine the overall shape of decision behaviors. Second, the result of Experiment 2 directly provides evidence against the model where the reference point is determined by the remaining number of opportunities. In terms of the remaining opportunities, the fourth opportunity in K = 5 condition and the first opportunity in K = 2 condition are identical. However, the empirical thresholds observed in the two cases are significantly different. The subjective optimality model, which assumes that the reference point is maintained over rounds, accounts for the data naturally.

**The assumption that the reference point is constant across rounds and problems is not convincing. An important feature of prospect theory is that the reference point can flexibly change depending on the decision context.**

We fully agree that the reference point can flexibly change depending on the context. In fact, the context-dependent reference points, and decision thresholds determined based on the differential reference points, are the core idea we tested in Experiment 2. Critical assumption we have made in the current study is how the ‘context’ is defined and how rapidly the context change would affect one’s reference point, and in turn, her/his valuation. Given the full information about the value distribution, we assumed that individuals would set an expectation about future outcomes and use the expectation as a reference point (Ericson & Fuster, 2011). Although the timescale of the ‘recent past’ experiences that affects individuals’ expectation is unspecified (Koszegi & Rabin, 2006, 2007), it is not uncommon to assume that individuals would apply the same adaptive valuation scheme within one block comprising a series of decision trials (e.g., Rigoli et al., 2016; Khaw et al., 2017; c.f., Barkan & Busemeyer, 2003). To clarify, we added the following paragraph stating the assumption we have made and its limitations.

[Page 10, Line 192] In the current study, we assume that individuals would set an expectation about future outcomes and use the expectation as a reference point [22].

[Page 11, Line 222] As described earlier, we assumed that individuals would set an expectation about future outcomes based on the number of opportunities they have, rather than trial-by-trial experience (c.f., [23]), and use the expectation as a reference point [22]. That is, we predicted that, based on our suggested model, one would set a lower (or higher) reference point in the context where one expects less (more) overall outcomes, which consequently lower (heighten) their decision thresholds.

[Page 20, Line 425] First, the reference point reflects each individual’s belief about the environment [14]. It is known that beliefs can alter how individuals respond to given information [24], and specifically in the current study, we hypothesized that different contexts (i.e., numbers of available opportunities) would change expectations about future outcomes, which in turn would alter individuals’ reference points [22]. In line with this, we showed that discouraged expectation (scarce opportunities in Experiment 2; K = 2) causes individuals to be more pessimistic about future chances and wait less in deciding (lowering thresholds). Moreover, encouraged expectation under abundant opportunities (K = 10 in Experiment 2) seemed like causing individuals to be more optimistic about future changes and wait longer.

Nevertheless, we further examined whether there are any hints that indicate dynamically changing reference points. First, to test the change of reference points along the course of the task (rounds), we divided task data into the first and second halves and compared whether or not the decision thresholds are different. Second, to test the change of reference points within each round across the opportunities, we ran logistic regressions examining the impact of preceding stimuli (numbers) on subsequent choices to accept or reject. In short, both tests did not show strong evidence suggesting the change of reference points. We have added the following results in Supporting Information.

[Page 22, Line 487] Nevertheless, we conducted two explorative analyses examining potential learning effects. First, each individual’s task data were divided into the first and the second half. Across all three experiments, decision thresholds estimated from the first and the second halves were comparable, indicating no sign of extensive learning (**Fig. S6**). Second, we ran a series of logistic regressions to examine the impact of presented stimulus at the preceding opportunity in subsequent choices. There was a slight hint of negative influence of preceding stimuli on subsequent choices, but the beta coefficients were less than 1/10 of the betas of the current stimulus (**Fig. S7**).

**Revision figure 12 (now added as figure S6 in SI).** Decision thresholds along the course of the task rounds.

**Revision figure 13 (now added as figure S7 in SI).** Effects of past stimuli values on the subsequent choice about the current value.

**3. The model explains the difference between Exp 1 (where the distribution is unknown) and Exp 2 (where the distribution is known) by fitting a higher reference point to Exp 1 data. However the difference between these experiments is a critical place where potential learning effects should be examined. As participants experience more rounds, they should build more accurate beliefs about the candidate’s distribution, which should shift the reference point in the model. The authors assumed the reference point was constant throughout the experiment, but it’d be reasonable to test this assumption by comparing reference points between the 1st half and the 2nd half.**

Thank you for the suggestion. Per the reviewer’s suggestion, potential effects of learning on decision behaviors were examined by separately estimating the thresholds in the first half (round 1 to round 100) and the second half (round 101 to round 200) of each experiment. We applied repeated measures ANOVA with the opportunity (1st, to K-1st) and the round (first half vs second half) as fixed factors, and participants as a random factor. In Experiment 1, the decision thresholds decreased from the first half to the second half and the change was statistically significant (F(1,19) = 14.4, p = 0.0012). However, the main effect of round was not statistically significant in Experiments 2 and 3. In Experiment 1, participants might have initially set the thresholds relatively high, and took the first half of the task adjusting their thresholds. This behavioral pattern in Experiment 1 is likely due to the fact that participants were not informed of the maximum value unlike the other experiments. The interaction effect between opportunity and round was not statistically significant (F(3,57) = 2.61, p = 0.060). In Experiment 2 (K=5) and Experiment 2 (K=10), neither the main effect of round nor the interaction effect was statistically significant. In Experiment 3, the interaction effect between the round and opportunity was statistically significant (F(3,60) = 3.35, p = 0.025). Note that the threshold at 4th opportunity in Experiment 3 tended to increase away from the optimal threshold value. This pattern suggests that the learning across rounds does not always move toward the optimal strategy but moves toward the thresholds that reflect the subjective value. The following figure as well as matching figure legend was added to Supporting Information.

**Revision figure 14 (now added as figure S6 in SI).** Decision thresholds along the course of the task rounds.

**4. The model explains the difference between Exps 1 and 2 (where decision criteria are too high in later rounds) and Exp 3 (where decision criteria are too low in early rounds) by including a “waiting cost” parameter that is only non-zero in Exp 3. This modeling solution feels ad hoc. How much time do the participants save (relative to a waiting cost of zero) and how much payment do they give up to save it?**

As described in the manuscript (Page 14, Line 290), the forced waiting time was implemented to temporally dissociate choice from other cognitive processes that are potentially reflected in pupil dilation data. However, when implementing the waiting time, we did hypothesize that the forced waiting time would increase mental cost (or sampling cost as previous literature describes; Costa & Averbeck, 2015; Lorenzen, 1981; Seale & Rapoport, 1997) of continuing to the later opportunities. The waiting cost estimated from our suggested computational model supported this hypothesis, such that the cost was larger in Experiment 3 compared to either Experiments 1 or 2.

To examine the actual impact of waiting cost, we simulated the expected number of choices and expected earnings with the best fitting parameters estimated in Experiment 3. The average number of choices across 200 rounds was 494 (SD: 53) and the average earning was 22961 KRW (SD: 233). When we repeated the same simulation after setting the waiting cost parameter zero, the average number of choices increased to 550 (SD: 31), and the average earning increased to 23122 KRW (SD: 141). On average, participants saved 56 trials (10% of all trials) at the cost of 161 KRW (less than 1% of earnings). It is difficult to measure the cost of time objectively. However, considering the relatively small loss in earnings, it seems plausible that participants chose to decrease the threshold, reducing 10% of trials at the cost of a very small portion of their future earnings.

**5. The pupil data largely replicate previous results showing an influence of choice and choice difficulty on pupil. That is, these data do not seem to be the major contribution of the paper, above and beyond the behavioral data and (potentially) modeling. The one novel finding here is an association between pupil dilation and value sensitivity. However, the authors write “This positive correlation between value sensitivity and pupil dilation was statistically significant at the threshold where decision difficulty is the highest” (p. 19, line 371-372). Were multiple comparisons corrected for in this analysis, and if so, how?**

The pupil dilation is now measured as the peak pupil dilation between the stimuli onset and 1500msec after the onset, and all the pupillometry results were edited accordingly. The association test and multiple comparison correction were conducted following these three steps. First, we smoothed each individual’s pupil dilation data along the threshold centered values (i.e., Value - Threshold _i-th_) by applying local regression using a 2D polynomial model. Second, the association between value sensitivity and pupil responses were calculated using Pearson’s correlation. Third, a cluster-based permutation method was used to correct for multiple comparisons (Maris et al., 2007).

**Revision figure 15 (now added as figure 5C).** Individuals who had higher value sensitivity in their estimated parameter (median split; red) showed more pronounced pupillometric responses reflecting the value information. Shades represent s.e.m.

**6. This is a minor point, but the decision problem and what participants actually know about the problem in each experiment needs to be explained more clearly at the outset of the results section. (Eg, do they know it’s a uniform distribution, do they know the max of the distribution, do they know the number of rounds, do they know the number of problems, do they know the payoff function, etc). All of this information can be gleaned from the methods, but this section is at the end of the manuscript, and this information is critical to understanding the modeling and the differences across experiments.**

Thank you for the suggestion. We added all relevant information to the initial part of each corresponding result section. All changes are copied below.

[Page 7, Line 137] In Experiment 1, participants had up to five opportunities within each round to examine a presented number and make choices either to accept or reject.

[Page 7, Line 140] Note that here in Experiment 1, participants were not explicitly informed of the distribution (including the maximum) from where the numbers were extracted.

[Page 11, Line 230] For all three separate task conditions (K = 2, 5, and 10), participants were informed that the maximum possible number that would be presented was 150, and any numbers between 0 and 150 (inclusive) were equally likely to appear.

[Page 14, Line 300] Participants had up to five opportunities per each round, and all other experimental settings were equal to Experiment 2 (see Methods for details).

→ Participants had up to five opportunities per each round, and all other experimental settings were equal to Experiment 2; the full distribution information was informed to participants, and all participants played up to 200 rounds (see Methods for details).

**Reviewer #3: This paper developed a computational model of subjective valuation to explain suboptimal behavior in finite sequential decision making contexts. The authors were interested in testing and justifying their model, including a physiological basis for their explanation. I commend the authors for pursuing this timely and important question.**

**Nevertheless, there is room for significant improvement in clarifying the authors’ claims. While the experimental results are compelling, the authors should establish much better context for their claims. Secondly, this affects the extent to which they can generalize their results. These concerns are outlined below, and I hope the authors can use this feedback to improve their manuscript.**

**Major Comments**

**1) The introduction does not establish sufficient context for their experiments. It is unclear a) What other explanations/models there are for the secretary problem, b) Why subjective valuation may be a good model to explain this behavior, c) What link pupillometry has to subjective value?**

We regret that the introduction did not provide sufficient context. Per the reviewer’s suggestion, we added the following paragraphs clarifying each commented point.

a) What other explanations/models there are for the secretary problem:

[Page 5, Line 78] Since the 1960’s when the secretary problem was originally introduced, a vast amount of studies were conducted examining the optimal solution of the problem [25-28]. Although an equally large volume of studies followed focusing on biases in human choice patterns, the major stream took a descriptive approach explaining the extent to which human choices drifted apart from the optimal solution [16, 29-31], leaving where the bias originated from an open question.

In various psychological studies, including an optimal stopping problem in the mate choice domain [11] and consumers’ purchasing decisions [12, 13], it was suggested that individuals’ heuristic valuation, the framework of Prospect theory [14], underlies the biases observed in their decision patterns [15]. Specifically, the Prospect theory has shown that individuals’ choices are guided by subjective valuation of potential gains and losses relative to the context where they are situated [14]. We constructed a computational model that adopts this concept of ‘reference point’ and examined whether the process of subjective valuation explains how individuals’ decision processes deviate from the optimal decision strategy. In addition, to capture individual differences in subjective valuation, we hypothesized that nonlinear value sensitivity (i.e., concave value function for gains and convex value function for losses) [14] and waiting cost [16, 17] parameters would take crucial parts in determining individuals’ decision patterns (see **Methods** for detailed model structure).

b) Why subjective valuation may be a good model to explain this behavior:

The optimal solution of the secretary problem is calculated under the assumption that individuals may set decision thresholds based on expected values of subsequent opportunities. The fact that empirical human behavioral choices deviated from the optimal solution as well as that there are quite a broad variability in their decision thresholds suggest the existence of individual differences in their valuation: i.e., usage of subjective valuation. As described in the manuscript, we formalized this subjective valuation using the framework of Prospect theory, of which explanatory power of biases in human choice patterns was repetitively shown in various psychological studies (Ruggeri et al., 2020). We hypothesized that such simple individual differences in subjective valuation may explain not only the decision thresholds within one task design, but also the systematic changes that occur between contexts.

c) What link pupillometry has to subjective value:

In the current study, pupil responses were used as a neurophysiological confirmatory measure of our suggested computational model. To clarify what link pupillometry has to subjective valuation, we added the following paragraph in the Introduction section that briefly reviews the findings reported in previous studies:

[Page 6, Line 109] In conjunction with the computational modeling approach, we recorded individuals’ pupil dilation responses and analyzed their association with participants’ choice behaviors to corroborate our suggested model. A rich set of evidence suggests that pupil dilation (or contraction)—a neurophysiological signature coinciding with norepinephrine levels in the brain—reflects not only individuals’ arousal level [1, 2], but also cognitively complex information, such as value [3, 4], uncertainty [1], surprise [5], cognitive conflict [6, 7], and choice [8]. More specifically, recent studies showed that pupil-linked neuromodulatory systems have a critical role in decision formation, rather than simply in reflection of the decision consequences [8], and moreover that the size of pupil responses is associated with individuals’ choice patterns [9, 10].

Based on these previous studies, we hypothesized that individuals’ pupil responses would be linked to their decision processes in three folds; i) pupil responses may be larger to the options that will be accepted versus rejected, ii) pupil dilation may reflect the deviation between option values and decision thresholds, supporting value- and threshold- based decision processes, and iii) pupil responses in individuals who show larger value sensitivity may be larger.

**2) Given the experimental design, K is a critical independent variable and it is unclear how the experimenters defined it at K=2 and K=5. What happens if K is increased to 10, 20, or 100? At minimum, K needs to be explained and justified more completely. The issue is that it is currently unclear how the results between Experiment 1, 2, and 3 build on each other. Especially going from experiment 2 to 3, it remains unclear whether the results are due to careful manipulations, or rather due to unintentional confounds introduced by virtue of changing the task design. Additional behavioral data could go a long way toward strengthening the authors’ claims here and improving the manuscript, but it may alternatively simply require expanded rationale and justification in the main text.**

As the reviewer noted, K indeed is a critical variable to the task design. Its effect was meant to be tested within Experiment 2, but we agree that our choice of K = 2 and 5 would seem quite arbitrary. We understand that diverse variants of the experimental conditions are possible. However, as an attempt to find basic behavioral patterns and decision mechanisms, we considered the following practical constraints.

First, we chose K = 5 as the baseline decision context i) to benchmark previous experimental studies (e.g., Guan et al., 2015, 2018), and ii) to provide the total number of opportunities that is small enough to obtain enough choice data for all opportunities in each individual and at the same time, large enough to reliably observe the changes in decision thresholds.

Second, we chose K = 2 as the decision context where there is only one valid opportunity decision-makers’ decision threshold determines the choice. As described in the main text, we used this task context as the context where participants had scarce opportunities (one opportunity to decide accept or reject per round; at the second opportunity, they always have to accept).

We agree that testing these two conditions may not be enough to generalize our model, and thus conducted an additional experiment where K = 10. This task condition provides more abundant opportunities to participants and gives us a chance to examine where our model-based prediction approach captures all three task contexts (K = 2, 5, and 10). The results were consistent with our expectation and well explained by our suggested model; individuals who participated in the context where they had abundant opportunities showed a higher reference point and accordingly, a higher decision threshold. The result section and figures related to Experiment 2 are edited now to include all three task contexts.

**Revision Figure 16 (now added as figure 3B and 3C).** Model-based threshold prediction using the changes in reference point; model-based threshold prediction matched with empirical data as well as with directly estimated model parameters.

Experiment 1 and 3 should be considered independently from Experiment 2. For both experiments, we set K = 5 because of the same reason we explained above. Experiment 1 indeed was the first experiment where we tested with a more naturalistic environment where participants were not informed of the maximum stimulus value. Experiment 3 was tailored to examine pupil responses under the condition where all other task features were kept the same (K = 5; full information). It was inevitable that longer forced waiting time embedded in each opportunity would be perceived more costly. Consistent with our expectation as well as previous literature about sampling cost (Costa & Averbeck, 2015; Lorenzen, 1981; Seale & Rapoport, 1997), the estimated waiting cost parameter was indeed larger than zero and larger than that of Experiment 2 under K = 5. We now added the following paragraphs in the introduction section to clarify our motivation for each task design.

[Page 6, Line 109] In conjunction with the computational modeling approach, we recorded individuals’ pupil dilation responses and analyzed their association with participants’ choice behaviors to corroborate our suggested model. A rich set of evidence suggests that pupil dilation (or contraction)—a neurophysiological signature coinciding with norepinephrine levels in the brain—reflects not only individuals’ arousal level [1, 2], but also cognitively complex information, such as value [3, 4], uncertainty [1], surprise [5], cognitive conflict [6, 7], and choice [8]. More specifically, recent studies showed that pupil-linked neuromodulatory systems have a critical role in decision formation, rather than simply in reflection of the decision consequences [8], and moreover that the size of pupil responses is associated with individuals’ choice patterns [9, 10].

Based on these previous studies, we hypothesized that individuals’ pupil responses would be linked to their decision processes in three folds; i) pupil responses may be larger to the options that will be accepted versus rejected, ii) pupil dilation may reflect the deviation between option values and decision thresholds, supporting value- and threshold- based decision processes, and iii) pupil responses in individuals who show larger value sensitivity may be larger. To test these hypotheses, we conducted an additional experiment (Experiment 3) where actions (choosing to accept or reject) were temporally dissociated from physiological responses to stimuli; participants (N = 24, male/female = 12/12, age = 22.67 ± 2.28 years) were not allowed to make a choice until an audio cue was played. All the other settings were equal to Experiment 2 where participants had up to five chances (K = 5). Non-overlapping samples were obtained from each experiment (see **Methods** for detailed procedures).

**3) The authors claim that Experiment 1 and 2 are in line with affecting neural hypotheses, whereas in fact these are purely behavioral results. This should be**

**developed further in the Introduction and in the description of the results.**

We regret that the description was somewhat misleading. The point we meant to emphasize from Experiments 1 and 2 is the impact of individuals’ beliefs on their behavioral choices, rather than its relevance to neural responses. To avoid any further confusion, we edited the sentence as follows.

[Page 20, Line 426] It is known that beliefs can alter how individuals respond to given information, which not only affects their behavioral choices, but also neural responses (24).

→ It is known that beliefs can alter how individuals respond to given information [24], and specifically in the current study, we hypothesized that different contexts (i.e., numbers of available opportunities) would change expectations about future outcomes, which in turn would alter individuals’ reference points [22].

**Minor Comments**

**1) An alternative explanation for the pupillometry results is that these systems are acting upon stress/norepinephrine networks. Notably, some studies have indicated that fluctuations in pupil diameter are associated with BOLD changes in the locus coeruleus (e.g., Murphy et al., 2014, HBM).**

We thank the reviewer for the comment. We cited previous studies including Murphy et al. (2014) to mention potential neural mechanisms that underlie pupil responses during the task.

[Page 21, Line 462] The pupil responses may be reflecting the involvement of the neuromodulatory systems (e.g., locus coeruleus [32]) in decision processes through release of norepinephrine, which temporarily facilitates computations in cortical networks [4, 8]. During the process of decision-making, individuals who had higher value sensitivity showed exaggerated pupil responses reflecting both decision difficulty and subsequent choice information. Consistent with previous studies, our pupillometry results evidence the association between neurophysiological pupil responses and individuals’ characteristics in valuation [9].

Alternatively, the observed pupil responses could be a result of top-down control, such that individuals may pay more attention to the stimuli that they plan to accept for accumulating more evidence [33] or a process following the opposite causality, in that the rich amount of accumulated evidence of a particular stimulus may induce even higher attention levels (e.g., saliency driven bottom-up attention [34]). In the current study, the latter is unlikely, given that all low-level visual information (e.g., contrast) of the displayed stimuli were matched or controlled for. To sum up, the current results show that the two pieces of information essential in subjective valuation are linked together at the physiological level, deeply involved in the process of decision formation [8].

**2) Among other limitations to external validity in the secretary problem include that these designs fail to constrain a decision-makers opportunity costs of time and computational capacity. As a result, it is confounded whether a decision-maker is suboptimal in a more realistic setting or is in fact optimizing factors that are more important to them (e.g., undersampling a pool of candidates because it requires less work, so that the team leader can focus on a project deadline). This issue could be considered more thoroughly in the Discussion section.**

Given the nature of sequential decision-making, as the reviewer pointed out, decision-makers’ concern about the opportunity cost always coincides with their other decision processes (e.g., noisy choices due to the limitation of their computational capacity). We believe that our computational modeling approach in conjunction with experiments under various contexts provided a chance to answer the involvement of each factor; waiting cost parameter corresponds to the opportunity cost and decision variability corresponds to the noisiness. In Experiment 1 where the decision-making context was relatively more realistic (participants do not know the maximum value of potential stimuli), estimated parameters revealed that participants showed more noisy decision patterns (larger decision variability) and that no evident effect of waiting cost existed (waiting cost not different from zero). Of course, we agree that these conclusions are yoked with the model structure we hypothesized. To open the question of expanding our computational model to capture more realistic setting, we added the following description in the discussion section.

[Page 22, Line 480] The future direction of the current study includes expanding our model further, so that it can capture more realistic decision contexts where various factors may interact (e.g., confidence, concerns about sunk cost, limitation of information processing capacity).

**3) Related to the point about the Introduction, please establish a link between behavioral value sensitivity and subjective value and the relationship of pupillometry to these concepts.**

We thank the reviewer for the suggestion. The following paragraphs are added to briefly review the link between pupillometry and subjective valuation, and to clarify our hypotheses that specifically point at the link.

[Page 6, Line 109] In conjunction with the computational modeling approach, we recorded individuals’ pupil dilation responses and analyzed their association with participants’ choice behaviors to corroborate our suggested model. A rich set of evidence suggests that pupil dilation (or contraction)—a neurophysiological signature coinciding with norepinephrine levels in the brain—reflects not only individuals’ arousal level [1, 2], but also cognitively complex information, such as value [3, 4], uncertainty [1], surprise [5], cognitive conflict [6, 7], and choice [8]. More specifically, recent studies showed that pupil-linked neuromodulatory systems have a critical role in decision formation, rather than simply in reflection of the decision consequences [8], and moreover that the size of pupil responses is associated with individuals’ choice patterns [9, 10].

Based on these previous studies, we hypothesized that individuals’ pupil responses would be linked to their decision processes in three folds; i) pupil responses may be larger to the options that will be accepted versus rejected, ii) pupil dilation may reflect the deviation between option values and decision thresholds, supporting value- and threshold- based decision processes, and iii) pupil responses in individuals who show larger value sensitivity may be larger. To test these hypotheses, we conducted an additional experiment (Experiment 3) where actions (choosing to accept or reject) were temporally dissociated from physiological responses to stimuli; participants (N = 24, male/female = 12/12, age = 22.67 ± 2.28 years) were not allowed to make a choice until an audio cue was played. All the other settings were equal to Experiment 2 where participants had up to five chances (K = 5). Non-overlapping samples were obtained from each experiment (see **Methods** for detailed procedures).

**4) It is not clearly justified how decision difficulty and evidence accumulation are associated with the subjective value model. How would response time factor into these models and the behavior? Could an alternative model employ the drift diffusion model to assess changes in valuation (e.g., Hutcherson et al., 2015, Neuron)?**

As per the signal detection theory, decision difficulty is defined as the distance from each choice option to the decision criteria. Such definition can be equally applied to the drift diffusion model and other models that hypothesize evidence accumulation. In brief, value-based decision-making process can be described as a dynamically evolving signal of “relative decision value” (Hutcherson et al., 2015, Neuron; Krajbich et al., 2015, Plos Comp Biol) where for example, a piece of unit evidence is defined as the value difference between options when the choice is between two alternatives. In a decision-making model as suggested in the current study, the value of options are assumed to be evaluated subjectively. Thus, if we were to use a modeling approach in the level of evidence accumulation (e.g., drift diffusion model), such subjective valuation should be considered as criteria that alter the size of unit evidence. In this perspective, naturally, choice patterns including response time can be explained as the output of the evidence accumulation process.

As explained above, it is inevitable that evidence accumulation is defined in extension of the subjective valuation model, and thus, such an evidence accumulation model cannot be considered as an alternative model. More importantly, the two models explain decision processes at different levels, and it would not be a fair comparison to set one next to the other model in parallel. We fully agree and have no doubt that sequential sampling models (e.g., drift diffusion model) would be most useful in a future study in investigating temporal evolution of value throughout each decision trial.

**5) The authors should explain why their experimental design is inconsistent with many results in the secretary problem, where other researchers have found that participants tend to undersample a pool of information.**

A recent study by Furl et al (2019) also reported a choice pattern where participants oversampled, rather than undersample, a pool of information. Among the potential factors why they think they observed the opposite pattern (e.g., usage of naturalistic image stimuli), Furl et al. (2019) suspected that a sampling incentive being larger than a sampling cost might underlie participants’ motivation to oversample. Although Furl et al. (2019) reported that this sampling reward model does not match their empirical data, our results across the three experiments were consistent with this perspective. Specifically, individuals showed higher thresholds for both Experiments 1 and 2 (oversampling), but lower thresholds for Experiment 3 (undersampling). The main change in Experiment 3 was the additional forced wait introduced before the cue when participants were allowed to submit their choice. Our model-based analysis results suggest that this subtle change in task design may have triggered participants to think more about the tradeoff between payoffs and time they spent per round. Such an impact of additional ‘cost of waiting (extra time)’ is consistent with previous reports showing that non-zero interview cost was associated with lowering decision thresholds (8, 15, 39, 40). Our model parameter estimates supported this interpretation, such that only in Experiment 3, the estimated cost was significantly larger than zero. To clearly deliver this interpretation in line with previous reports, we added the following paragraph to the discussion section.

[Page 19, Line 404] However, a recent study reported the opposite pattern, i.e., higher decision thresholds compared to the optimal, in a variant of optimal stopping problem, and suggested a possibility that choice biases may occur in both direction [11]. Among the potential factors why they think they observed the opposite pattern (e.g., usage of naturalistic image stimuli), Furl et al. [11] suspected that a sampling incentive being larger than a sampling cost might underlie participants’ motivation to oversample. Although Furl et al. [11] reported that this sampling reward model does not match their empirical data, our results across the three experiments were consistent with this perspective.

**6) The GitHub link for the code/data is not working. Please check.**

We activated the GitHub link (<https://github.com/dongilchung/secretary-problem>) and uploaded data and codes for the current study.

**References**

1.	Urai AE, Braun A, Donner TH. Pupil-linked arousal is driven by decision uncertainty and alters serial choice bias. Nature communications. 2017;8(1):1-11.

2.	Nassar MR, Rumsey KM, Wilson RC, Parikh K, Heasly B, Gold JI. Rational regulation of learning dynamics by pupil-linked arousal systems. Nature neuroscience. 2012;15(7):1040.

3.	Van Slooten JC, Jahfari S, Knapen T, Theeuwes J. How pupil responses track value-based decision-making during and after reinforcement learning. PLoS computational biology. 2018;14(11):e1006632.

4.	Einhauser W, Koch C, Carter O. Pupil dilation betrays the timing of decisions. Frontiers in human neuroscience. 2010;4:18.

5.	Preuschoff K, t Hart BM, Einhauser W. Pupil dilation signals surprise: Evidence for noradrenaline’s role in decision making. Frontiers in neuroscience. 2011;5:115.

6.	Cavanagh JF, Wiecki TV, Kochar A, Frank MJ. Eye tracking and pupillometry are indicators of dissociable latent decision processes. Journal of Experimental Psychology: General. 2014;143(4):1476.

7.	Critchley HD, Tang J, Glaser D, Butterworth B, Dolan RJ. Anterior cingulate activity during error and autonomic response. Neuroimage. 2005;27(4):885-95.

8.	de Gee JW, Knapen T, Donner TH. Decision-related pupil dilation reflects upcoming choice and individual bias. Proceedings of the National Academy of Sciences. 2014;111(5):E618-E25.

9.	Eldar E, Felso V, Cohen JD, Niv Y. A pupillary index of susceptibility to decision biases. Nature Human Behaviour. 2021:1-10.

10.	Keung W, Hagen TA, Wilson RC. Regulation of evidence accumulation by pupil-linked arousal processes. Nature Human Behaviour. 2019;3(6):636-45.

11.	Furl N, Averbeck BB, McKay RT. Looking for Mr (s) Right: Decision bias can prevent us from finding the most attractive face. Cognitive psychology. 2019;111:1-14.

12.	Zhou C, Tang W, Zhao R. Optimal consumer search with prospect utility in hybrid uncertain environment. Journal of Uncertainty Analysis and Applications. 2015;3(1):1-20.

13.	Kogut CA. Consumer search behavior and sunk costs. Journal of Economic Behavior & Organization. 1990;14(3):381-92.

14.	Kahneman D, Tversky A. Prospect theory: An analysis of decision under risk. Econometrica: Journal of the Econometric Society. 1979:263-91.

15.	Ruggeri K, Alí S, Berge ML, Bertoldo G, Bjørndal LD, Cortijos-Bernabeu A, et al. Replicating patterns of prospect theory for decision under risk. Nature human behaviour. 2020;4(6):622-33.

16.	Seale DA, Rapoport A. Sequential decision making with relative ranks: An experimental investigation of the" secretary problem". Organizational behavior and human decision processes. 1997;69(3):221-36.

17.	Lorenzen TJ. Optimal stopping with sampling cost: the secretary problem. The Annals of Probability. 1981;9(1):167-72.

18.	Mosteller F, Nogee P. An experimental measurement of utility. Journal of Political Economy. 1951;59(5):371-404.

19.	Konovalov A, Krajbich I. Revealed strength of preference: Inference from response times. Judgment & Decision Making. 2019;14(4).

20.	Clithero JA. Response times in economics: Looking through the lens of sequential sampling models. Journal of Economic Psychology. 2018;69:61-86.

21.	Sharot T, Korn CW, Dolan RJ. How unrealistic optimism is maintained in the face of reality. Nature neuroscience. 2011;14(11):1475.

22.	Marzilli Ericson KM, Fuster A. Expectations as endowments: Evidence on reference-dependent preferences from exchange and valuation experiments. The Quarterly Journal of Economics. 2011;126(4):1879-907.

23.	Barkan R, Busemeyer JR. Modeling dynamic inconsistency with a changing reference point. Journal of Behavioral Decision Making. 2003;16(4):235-55.

24.	Paulus MP, Angela JY. Emotion and decision-making: affect-driven belief systems in anxiety and depression. Trends in cognitive sciences. 2012;16(9):476-83.

25.	Freeman P. The secretary problem and its extensions: A review. International Statistical Review/Revue Internationale de Statistique. 1983:189-206.

26.	Hill TP, Krengel U. Minimax-optimal stop rules and distributions in secretary problems. The Annals of Probability. 1991;19(1):342-53.

27.	Bearden JN. A new secretary problem with rank-based selection and cardinal payoffs. Journal of Mathematical Psychology. 2006;50(1):58-9.

28.	Gilbert JP, Mosteller F. Recognizing the maximum of a sequence. Selected Papers of Frederick Mosteller: Springer; 2006. p. 355-98.

29.	Goldstein DG, McAfee RP, Suri S, Wright JR. Learning when to stop searching. Management Science. 2020;66(3):1375-94.

30.	Guan M, Lee MD. The effect of goals and environments on human performance in optimal stopping problems. Decision. 2018;5(4):339.

31.	Lee MD. A hierarchical bayesian model of human decision‐making on an optimal stopping problem. Cognitive science. 2006;30(3):1-26.

32.	Murphy PR, O'connell RG, O'sullivan M, Robertson IH, Balsters JH. Pupil diameter covaries with BOLD activity in human locus coeruleus. Human brain mapping. 2014;35(8):4140-54.

33.	Krajbich I, Armel C, Rangel A. Visual fixations and the computation and comparison of value in simple choice. Nature neuroscience. 2010;13(10):1292.

34.	Koch C, Ullman S. Shifts in selective visual attention: towards the underlying neural circuitry. Matters of intelligence: Springer; 1987. p. 115-41.
