# Supplementary material for: Subjective optimality in finite sequential decision-making
Source: PLoS Comput Biol. 2021 Dec 16;17(12):e1009633. doi: 10.1371/journal.pcbi.1009633 (PMC8675647; doi:10.1371/journal.pcbi.1009633)
Supplement: Attachment — Submitted filename: Response_to_reviewers_R2-submit.docx [file pcbi.1009633.s003.docx]

Dear Dr. Gershman and Dr. Haith,

Thank you very much for considering our manuscript PCOMPBIOL-D-20-01425R (“Subjective optimality in finite sequential decision-making”) for publication in PLOS Compuational Biology. We appreciate the opportunity to revise and resubmit our manuscript once more. Following the suggestion from the editors and reviewers, we now tried to clearly show the specific features of behavior which our suggested model, but not other models, successfully explain and simulate. In addition, we included a more complete discussion of alternative models to our revised manuscript. We believe that the current revision helped improve delivery of the strengths that our suggested computational model has over existing models.

The reviewers’ comments are fully quoted below in bold; our responses follow each comment. Changes to the submission in response to the  reviewers’ comments are highlighted in blue in the revised manuscript and supplementary materials and also reproduced or summarized below.

**--**

**Dear Dr Chung,**

**Thank you very much for submitting your manuscript "Subjective optimality in finite sequential decision-making" for consideration at PLOS Computational Biology.**

**As with all papers reviewed by the journal, your manuscript was reviewed by members of the editorial board and by several independent reviewers. The revision addressed many of the previous issues raised by the reviewers. However, the reviewers highlighted a number of concerns that still remain. Of particular importance, it is not clear whether the model proposed in the paper provides a better account of the data, or even can be distinguished from, alternative models - especially the linear threshold model proposed in Baumann et al., 2020. The revised paper includes a model comparison, however this appears to show little or no advantage of the subjective valuation model over the linearly declining threshold model.**

**Although the paper introduces a novel and interesting perspective on the stopping problem, in order to meet the standard for PLOS Computational Biology we feel it is important to demonstrate that the proposed model can explain aspects of behavior that are not already accounted for by existing models. Model comparison is one way to establish this. However an alternative approach, rather than comparing the quality of global fits, might be to identify and test specific features of behavior by which the models should be expected to differ.**

**In light of this issue, we would like to invite the resubmission of a significantly revised version, provided you can provide clear evidence in favor of the subjective optimality model over alternative models. The revised paper should also include a more complete discussion of alternative models, as well as taking into account the reviewers' other comments.**

**We cannot make any decision about publication until we have seen the revised manuscript and your response to the reviewers' comments. Your revised manuscript is also likely to be sent to reviewers for further evaluation.**

**When you are ready to resubmit, please upload the following:**

**[1] A letter containing a detailed list of your responses to the review comments and a description of the changes you have made in the manuscript. Please note while forming your response, if your article is accepted, you may have the opportunity to make the peer review history publicly available. The record will include editor decision letters (with reviews) and your responses to reviewer comments. If eligible, we will contact you to opt in or out.**

**[2] Two versions of the revised manuscript: one with either highlights or tracked changes denoting where the text has been changed; the other a clean version (uploaded as the manuscript file).**

**Important additional instructions are given below your reviewer comments.**

**Please prepare and submit your revised manuscript within 60 days. If you anticipate any delay, please let us know the expected resubmission date by replying to this email. Please note that revised manuscripts received after the 60-day due date may require evaluation and peer review similar to newly submitted manuscripts.**

**Thank you again for your submission. We hope that our editorial process has been constructive so far, and we welcome your feedback at any time. Please don't hesitate to contact us if you have any questions or comments.**

**Sincerely,**

**Adrian M Haith
Associate Editor
PLOS Computational Biology**

**Samuel Gershman
Deputy Editor
PLOS Computational Biology

*************************

**Reviewer's Responses to Questions**

**Comments to the Authors:**

**Reviewer #1: The authors have done quite some work to address all the comments from the previous round of reviews. The paper is certainly improved. However, I am still not convinced that there is no selection bias here, nor am I convinced about the evidence in favor of the Prospect Theory (PT) model. These issues are linked. Because of the potential selection bias, it is unclear how much the thresholds really differ from “optimal”, and that is the crux of the argument for the PT model.**

**(1) The authors counter-argue that the threshold estimation was done at the individual level and therefore selection bias is not an issue. There is a problem with this argument, in that it assumes that all the variability in thresholds is at the subject level. But what if there is variability within-subject across trials? There is always some variability, the question is just how much. With any amount of variability, trials with low thresholds will disappear at earlier opportunities, leaving the higher threshold trials for later opportunities. It certainly seems like this could account for many of the patterns in the data. For instance, it is notable that there does not appear to be any deviation from optimality at the 1st opportunity. It is also notable that the deviation from optimality increases across opportunities. Both of these would I think be predicted by selection bias.**

**Another key set of results is on page 12, where the authors compare the threshold at 1/2, 4/5 and 9/10. There is clearly a difference between 1/2 and the others (which could easily be attributable to selection bias), but the difference between 4/5 and 9/10 is quite small, and seemingly non-significant (though the authors don’t report that comparison). So, overall the evidence here is not very compelling.**

**Response:** Thank you for detailing your concern. We now ran a new simulation to implement how the behavioral pattern would look if the selection bias indeed is the major factor that would bias individuals away from the optimal behavioral pattern.

As the reviewer pointed out, one of the sources of potential variability in individuals’ decision thresholds is within-subject variability that affects individuals’ across-round behaviors. To run the simulation, we calculated the behavioral choice variability from the entire data (across- opportunities and rounds), and assumed it all would contribute to the within-subject variability across rounds (i.e., there is no across-opportunity variability within a round). This is the most conservative setting, because large across-round variability opens up the possibility that only the rounds in which decision thresholds happened to be high contribute to the post-hoc estimation of the later opportunities; in turn, as the reviewer noted, this would make the estimated decision thresholds to have a shallower slope (more flat) across opportunities compared to the optimal decision model.

As illustrated in the Response figure 1, the Selection bias model can partially explain the deviation from the optimal thresholds and show relatively shallower slopes as expected. However, compared to empirical decision thresholds across opportunities, the simulation slopes were relatively steeper even though the simulation was run with the maximum level of variability as we described above. We acknowledge and would like to clarify that the distinctive deviation at the initial opportunities in Experiment 3 between the Selection bias model and empirical data should not be used as the evidence against the potential impact of selection bias, and thus added an explicit description about its interpretation for readers.

**Response figure 1 (now added as figure S12A in SI).** Decision thresholds simulated with a selection bias model are compared against the optimal decision model and empirical data.

Although our simulation using the maximum level of within-subject variability across rounds was our attempt to give the Selection bias model the maximal power to explain “flat” decision thresholds across opportunities, we additionally examined the impact of different levels of within-subject variability on decision thresholds. There was a stark difference between the simulated and empirical data when probed through the association between within-subject variability across rounds and decision thresholds. As illustrated in Response figure 2 and consistent with our description above, the largest response variability in the Selection bias model was associated with the smallest threshold change across opportunities, and vice versa (r = -0.97, *P* = 1.70e-40). On the contrary, empirical data revealed that participants who had the largest response variability showed the largest threshold change across opportunities (r = 0.43, *P* = 5.21e-04), which is the exact opposite of the Selection bias model. These results suggest that selection bias cannot explain empirical decision patterns observed in this task.

**Response figure 2 (now added as figure S12B in SI).** Steepness of the changes in decision thresholds as a function of simulated response variability shows a different pattern from that of empirical data.

We added the aforementioned simulation results in Supporting Information, and also edited the main manuscript to include the originally omitted post-hoc comparison statistics between decision thresholds (e.g., 1/2, 4/5, versus 9/10). Edits we made are duplicated below:

[Page 3, Supporting Information]

**Selection biases.** It is possible that due to decision variabilities, participants’ choice rounds where they happened to have lower decision thresholds would be accepted at earlier opportunities, and thus be under-represented at subsequent opportunities. We ran a new simulation to implement how the behavioral pattern would become if the selection bias indeed is the major factor that biases individuals away from the optimal behavioral pattern.

One of the sources of potential variability in individuals’ decision thresholds is within-subject variability that affects individuals’ across-round behaviors. To run the simulation, we calculated the behavioral choice variability from the entire data (across- opportunities and rounds), and assumed it all would contribute to the within-subject variability across rounds (i.e., there is no across-opportunity variability within a round). This is the most conservative setting, because large across-round variability opens up the possibility that only the rounds in which decision thresholds happened to be high decision thresholds to contribute to the post-hoc estimation of the later opportunities; in turn, this would make the estimated decision thresholds to have a shallower slope (more flat) across opportunities compared to the optimal decision model.

As illustrated in **figure S12A**, the Selection bias model can partially explain the deviation from the optimal thresholds and show relatively shallower slopes as expected. However, compared to empirical decision thresholds across opportunities, the simulation slopes were relatively steeper even though the simulation was run with the maximum level of variability as we described above. Note that in this simulation-based analysis, we are using the differences in slopes across decision thresholds, rather than other features (e.g., the deviation at the initial opportunities in Experiment 3), as criteria to examine whether the simulation model captures the core mechanism explaining the empirical data.

Although our simulation using the maximum level of within-subject variability across rounds was our attempt to give the Selection bias model the maximal power to explain “flat” decision thresholds across opportunities, we additionally examined the impact of different levels of within-subject variability on decision thresholds. There was a stark difference between the simulated and empirical data when probed through the association between within-subject variability across rounds and decision thresholds. Consistent with our description above, the largest response variability in the Selection bias model was associated with the smallest threshold change across opportunities, and vice versa (r = -0.97, *P* = 1.70e-40; **Fig. S12B**). On the contrary, empirical data revealed that participants who had the largest response variability showed the largest threshold change across opportunities (r = 0.43, *P* = 5.21e-04), which is the exact opposite of the Selection bias model. Together, these results suggest that selection bias cannot explain empirical decision patterns observed in this task.

[Page 12, Line 252] ~ were significantly different depending on the number of opportunities one had per round (threshold_K=2, 1st_ = 79.28 ± 15.45, threshold_K=5, 4th_ = 90.42 ± 9.72, threshold_K=10, 9th_ = 93.50 ± 17.77; F(2,61) = 5.69, $\eta^{2}$ = 0.157, *p* = 0.0054; **Fig. S6**)

→ ~ were significantly different depending on the number of opportunities one had per round (threshold_K=2, 1st_ = 79.28 ± 15.45, threshold_K=5, 4th_ = 90.42 ± 9.72, threshold_K=10, 9th_ = 93.50 ± 17.77; F(2,61) = 5.69, $\eta^{2}$ = 0.157, *p* = 0.0054; post-hoc comparison K = 5 vs 2: CI = [0.49, 21.78], *p* = 0.038; K = 10 vs 2: CI = [3.44, 25.00], *p* = 0.0067; K = 10 vs 5: CI = [-7.93, 14.10], *p* = 0.78; **Fig. S8**)

[Figure S12] Subjective optimality model versus an alternative Selection bias model

Diminishing decision thresholds across opportunities alternatively may be driven by a combination of within-subject decision variability and selection bias where rounds in which decision thresholds happened to be higher (due to the within-subject variability across rounds) lasting further at later opportunities. (A) To directly examine this effect, decision thresholds simulated with a selection bias model are compared against the optimal decision model and empirical data. The Selection bias model can explain the observed empirical data fairly well for some of the parts in Experiments 1 and 2, but not all of the patterns (see Supplementary text). Error bars indicate s.e.m. (B) We additionally examined the impact of different levels of within-subject variability on decision thresholds. There was a stark difference between the simulated and empirical data when probed through the association between within-subject variability across rounds and decision thresholds. The largest response variability in the Selection bias model was associated with the smallest threshold change across opportunities, and vice versa (r = -0.97, *P* = 1.70e-40). On the contrary, participants who had the largest response variability showed the largest threshold change across opportunities (r = 0.43, *P* = 5.21e-04), which is the exact opposite of the Selection bias model. Each dot represents individual simulation.

**(2) Several of us asked for more evidence specifically in favor of the PT model. Here again the authors’ response was somewhat lacking. I do appreciate the model comparison, but the motivation and description of the new models was somewhat lacking; the entirety of it appears in a single supplemental figure. Moreover, the evidence isn’t that compelling. The advantage of the subjectively optimal model over the linear model is slight, and disappears once you remove the wait cost.**

**If we want to take this PT model seriously, shouldn’t it predict that people will be very unlikely to accept a number that is just below their threshold? Using the derived subjectively optimal thresholds for each subject, the authors could test whether there is a “gap” just below those thresholds where there are noticeably fewer acceptances than one would expect from symmetric noisy behavior around the thresholds. Ideally this would be done “out of sample” where the opportunity where this is tested is not used in fitting the subjectively optimal thresholds.**

**Response:** We apologize for the lack of clarity in our model descriptions. One part that we failed to clarify in the original manuscript is how the choices were implemented in relation to decision thresholds, which we believe is connected to the second part of the reviewer’s comment. In all the tested models, we introduced a gaussian noise around the threshold, which mirrors the stochastic nature of choices rather than assuming that choices are made deterministically before and after each threshold value (as applied in Signal detection theory). The width of the gaussian noise is set as a free parameter (described as a decision variability *s*) and estimated in individual-level. Given such a decision variability term, all models, not only our Subjective optimality model, predict that individuals’ tendency to accept *gradually* (reflecting the gaussian noise) decreases as a function of the distance between a presented number and the threshold. For better clarity, we made the following edits in the methods section:

[Page 33, Line 724] Here in all the tested models, we introduced a gaussian noise around computed decision thresholds, which mirrors the stochastic nature of choices rather than assuming that choices are made deterministically before and after each threshold value (as applied in Signal detection theory). The width of the gaussian noise is set as a free parameter (termed as a decision variability *s*) and estimated in individual-level.

Besides the aforementioned edit, we fully agree with the points both the reviewer and the editors raised about insufficient evidence for the Subjective optimality model over the linear model [1], and thus, further analyzed behavioral data to highlight a unique behavioral feature that only can be explained by our suggested model. Specifically, decision thresholds calculated from empirical data showed a decreasing pattern along the opportunities (Fig. 1b, 3a, 4b), but moreover, the extent of threshold change between opportunities also showed a decreasing pattern (i.e., second derivative of the decision threshold curve < 0). Such a qualitative difference between the linear threshold model and the empirical data was corroborated by the observed statistical difference across opportunities in all four experiments ([1^st^-2^nd^] vs [2^nd^-3^rd^] vs [3^rd^-4^th^]; Exp1: F(2, 38) = 14.13, η^2^ = 0.43, p = 2.59e-05; Exp2 (K=5): F(2, 40) = 20.10, η^2^ = 0.50, p = 9.06e-07; Exp2 (K=10): F(7, 119) = 3.46, η^2^ = 0.17, p = 0.0021; Exp3: F(2, 40) = 25.01, η^2^ = 0.56, p = 9.00e-08). As illustrated below in Response figure 3, such a change in the slope of decision threshold curve is a unique feature that our suggested Subjective optimality model successfully could explain (differences between the model and empirical data do not vary across opportunities; Exp1: F(2, 38) = 0.70, η^2^ = 0.036, p = 0.090; Exp2 (K=5): F(2, 40) = 2.37, η^2^ = 0.11, p = 0.11; Exp2 (K=10): F(7, 119) = 0.67, η^2^ = 0.039, p = 0.70; Exp3: F(2, 40) = 1.80, η^2^ = 0.082, p = 0.18), and has superior explanatory power to the linear threshold model.

**Response figure 3 (now added as figure S11 in SI).** The slope of decision threshold curve decreases along the opportunities. Negative values on the y-axis indicate that decision thresholds decrease between opportunities.

These new sets of data and corresponding descriptions are now added in the main text and Supporting Information as follows:

[Page 2, Supporting Information]

**Linear threshold model.** In a recent study, Baumann et al. [1] suggested that human stopping decisions in sequential decision-making can be well explained with a linear threshold model, which assumes a linear relationship among the thresholds across the opportunities (i.e., *Threshold*_i-th_ = a + b*i* where a and b are free parameters) [1]. In this Linear threshold model, a decision maker does not need to use dynamic programming approach.

One key feature that clearly dissociates the Linear threshold model from other models is the linear relationship in decision thresholds between opportunities. Besides examining its model fit, to examine whether the Linear threshold model well explains the empirical data, we further analyzed behavioral data and tested whether the data show such linear patterns. Specifically, decision thresholds calculated from empirical data showed a decreasing pattern along the opportunities (**Fig. 1b, 3a, 4b**), but moreover, the extent of threshold change between opportunities also showed a decreasing pattern (i.e., second derivative of the decision threshold curve < 0; **Fig. S11**). Such a qualitative difference between the linear threshold model and the empirical data was corroborated by the observed statistical difference across opportunities in all four experiments ([1^st^-2^nd^] vs [2^nd^-3^rd^] vs [3^rd^-4^th^]; Exp1: F(2, 38) = 14.13, η^2^ = 0.43, p = 2.59e-05; Exp2 (K=5): F(2, 40) = 20.10, η^2^ = 0.50, p = 9.06e-07; Exp2 (K=10): F(7, 119) = 3.46, η^2^ = 0.17, p = 0.0021; Exp3: F(2, 40) = 25.01, η^2^ = 0.56, p = 9.00e-08). These results suggest that individuals’ choice data in the current variant of Secretary problem task cannot be fully explained by the Linear threshold model.

[Page 21, Line 472]

The conclusions of the present study regarding the subjective valuation recruited in determining decision thresholds differ from previous findings by Baumann et al. [1], who argued that people rely on a mental heuristic and determine their thresholds linearly. As illustrated in our formal model comparison (**Fig. S1**; **Supplementary text**), individuals’ behavioral choices in each opportunity were explained by the Linear threshold model as well as by the Subjective optimality model. However, the decision patterns between opportunities were not accounted for by a linear decrease in decision thresholds, which is the main feature of the Linear threshold model (**Fig. S11**; **Supplementary text**). This discrepancy suggests that individuals’ choice data at least in the current variant of Secretary problem tasks cannot be fully explained by linear heuristic processes. Nevertheless, subtle differences in task designs (e.g., stimuli values followed a normal distribution in Baumann et al. [1], but a uniform distribution in the current study) may recruit different cognitive strategies, and thus future studies should explore potential factors that affect individuals’ decision processes (see **Supplementary text** and **Fig. S11-13** for further discussions).

[Figure S11] Subjective optimality model versus an alternative Linear threshold model (Baumann et al. [1])

Decision thresholds calculated from empirical data showed a decreasing pattern along the opportunities (Fig. 1b, 3a, 4b), but moreover, the extent of threshold change between opportunities also showed a decreasing pattern (i.e., second derivative of the decision threshold curve < 0). Such a change in the slope of decision threshold curve is a unique feature that our suggested Subjective optimality model successfully could explain (differences between the model and empirical data do not vary across opportunities; Exp1: F(2, 38) = 0.70, η^2^ = 0.036, p = 0.090; Exp2 (K=5): F(2, 40) = 2.37, η^2^ = 0.11, p = 0.11; Exp2 (K=10): F(7, 119) = 0.67, η^2^ = 0.039, p = 0.70; Exp3: F(2, 40) = 1.80, η^2^ = 0.082, p = 0.18), and has superior explanatory power to the linear threshold model. Negative values on the y-axis indicate that decision thresholds decrease between opportunities. Error bars indicate s.e.m.

**(3) I appreciate the RT analysis, but I think that for Figure 1c to be really compelling, the authors would want to compare to a version with the best-fitting constant threshold across opportunities. Otherwise it is difficult to judge whether these curves convincingly support dynamic over constant thresholds.**

**Response:** Following the reviewer’s suggestion, we redid the RT analysis with the best-fitting constant threshold across opportunities.

**Response figure 4.** Response time for each opportunity as a function of signed distance between the presented stimuli values and the thresholds estimated from the constant threshold model.

Note that the symmetric inverse V shape disappeared in this new figure, suggesting that response time for behavioral choices does not match with the choice difficulty defined based on the constant threshold model.

**(4) It seems that model-based threshold predictions for K=5 are missing? I think this is because that was the dataset where the model was trained. However, that could be remedied by repeating the same exercise using a different dataset to train the model on, e.g. training on K=10 and then predicting on K=2 and K=5.**

**Response:** As the reviewer noted, we did not include the model-based threshold predictions for K=5 because the predictions were made based on the corresponding empirical data. As suggested, we ran additional prediction analyses where we predicted K=2 and K=5 based on the estimated parameters from K=10 data. As depicted in Response figure 5, model-based predictions of the decision threshold using the K=5 data or the K=10 data as a reference showed comparable results.

**Response figure 5 (now added as figure S3 in SI).** Model-based predictions of the decision threshold made for K=2 and 10 using the K=5 data as a reference (left) showed comparable results with the prediction for K=2 and 5 using the K=10 data as a reference (right). Note that the confidence interval of the prediction (shaded area) is wider for the prediction based on K=10 data due to the wider variability in the original empirical data (red error bar for K=10).

These new sets of data and corresponding descriptions are now added in the main text and Supporting Information as follows:

[Page 13, Line 277]

The performance of model-based prediction showed comparable results when the empirical data in K = 10 were used to predict decision thresholds for K = 2 and 5 (**Fig. S3**).

[Figure S3] **Model-based prediction using K=10 data**

Model-based predictions of the decision threshold (blue) made for K=2 and 5 using the K=10 data as a reference showed comparable results with the prediction for K=2 and 10 using the K=5 data as a reference (**Fig. 3B**). Note that the confidence interval of the prediction (shaded area) is wider for the prediction based on K=10 data due to the wider variability in the original empirical data (red error bar for K=10). Error bars represent 95% confidence interval.

**(5) Regarding the pupil analyses: I think these are improved, but there is one lingering question. Do these analyses include the last opportunity, i.e. 5 out of 5? That decision seems fundamentally different than the others, since subjects should accept any number. There is still a small trend in RT here, but the decisions are noticeably faster overall, and asymptote pretty quickly above value = 0. I wonder if Opportunity = 5 is overrepresented in the “Value-Threshold > 0”, which could explain why the pupil size appears so flat for acceptances compared to rejections in Figure 5B. Basically, it would be helpful to see this figure broken down by opportunity number, five panels in total. That would help to further support the claim that pupil dilation is different for acceptances compared to rejections.**

**Response:** Thank you for this insightful comment. The original figure indeed included the last opportunity, and we agree that the last opportunity being qualitatively different from others might have some impacts on the average pupil pattern. We reran the same analysis in several different ways to examine whether the last opportunity is overrepresented in the pupil pattern. As depicted in Response figure 6, removing the data from the last opportunity did not have much impact on the overall pattern (compare Response figures 6A versus 6B). Illustration of each opportunity revealed that the overall patterns between values and pupil sizes are comparable across opportunities, except the pupil responses at opportunities 1 and 5 where stimuli values were near the threshold (near zero on the x-axis on Response figures 6C, 6D). Pupil responses at opportunity 5 were *not* flat and responsive to stimuli value, which indicates that participants actively engaged in evaluating the value even if the participants simply had to accept. On the contrary to pupil responses for accepted choices, pupil patterns for rejection choices were comparable across opportunities. We added the following figure and corresponding description in the revised Supporting Information.

**Response figure 6 (now added as figure S5 in SI). (A)** The peak pupil sizes are depicted as a function of the signed distance between stimuli value and the corresponding decision threshold. Given the nature of the last opportunity that individuals have no choice but to accept any stimuli values, pupil responses from **(B)** opportunities 1 to 4, **(C)** opportunity 1 separately from opportunities 2 to 4, and **(D)** opportunity 5 alone are illustrated in separate panels. Shades represent s.e.m.

[Page 16, Line 359]

A comparable result was observed across all the opportunities (**Fig. S5**) or ~

**Minor comments:**

**In what sense is Prospect Theory “heuristic”? It seems just as complex, if not more so, than expected utility. The authors might look at the recent work by Diederich & Trueblood (2018; Psychological Review) for one potential justification for this.**

**Response:** We simply adopted the term “heuristic” that was used in the earlier work of Tversky & Kahneman [2] where they summarized decision characteristics found in human decision-making. Particularly, the reference point introduced in Prospect theory, dissociating gains from losses, has been introduced as one of the heuristics (the impact of adjustment from an anchor) in their work. This is consistent with the take that was shown in Diederich & Trueblood [3] where the heuristic, intuitive, and fast process governed by ‘System 1’ was constructed according to Prospect theory.

**The authors might also be interested in Diehl & Poynor (2018), which shows that people have higher expectations when asked to choose from larger sets. They feel more disappointment for the same value item when it comes from a larger set than when it comes from a smaller set. This seems to parallel the current authors’ results.**

**Response:** Thank you for this suggestion. We agree that the suggested work parallels our results and used it to expand our discussion as follows:

[Page 20, Line 448] Such an increment of expectation in response to abundant opportunities parallels a recent study in marketing where they observed higher expectation in individuals’ satisfaction for the choices from larger than smaller set of assortments [4].

**Reviewer #2: I thank the authors for engaging with my previous comments and for the new analyses and revisions they have made to the manuscript. Unfortunately, my overall evaluation of the manuscript has not changed much. I continue to think that the main contribution is the novel empirical behavioral results, and to find the modeling less convincing.**

**1. Regarding my first comment on whether the assumptions of the dynamic programming modeling approach are justified, the new analyses suggest that there is not much learning happening, outside of experiment 1 where subjects do learn about the distribution of candidates, and/or that what learning does occur happens quickly. This is reassuring. However, the new analyses also show that there is an effect of previous candidate values (which were passed over) on current choices. The authors dismiss this finding as small, but the deviations from the optimal solution are also relatively small, and occur for exactly those decisions (3rd and 4th opportunities) where this is an effect of previous candidates. Therefore, it seems that a reasonable alternative (or addition) to subjective optimality model would that people are less likely to accept candidates that are above the optimal threshold but below a value that they’ve previously passed up. This kind of regret would result in subjects not adjusting their thresholds down as strongly as they should.**

**The model comparison also suggests that the subjective optimality model does not fit significantly better than a simple model that assumes people set a threshold and then linearly reduce that threshold as opportunities pass. It seems that such a heuristic model is another alternative to the subjective optimality model that cannot be ruled out by the data.**

**Response:** Thank you for this constructive comment. To more directly examine potential effects of “regret”, as suggested by the reviewer, we simulated behavioral choices with additional computational model where participants would accept candidates that are higher than both the optimal threshold and the highest value they have passed up in previous opportunities. To examine the maximum impact of past stimuli values, we applied the aforementioned rule of regret as deterministic decision thresholds at each opportunity and also assumed perfect memory (i.e., all the past stimuli values, rather than the most recent stimulus, affect decisions within the corresponding round). We simulated 10000 rounds of behavioral choices of pseudo-subjects, and estimated the decision thresholds and the effects of past values on the current decision. These steps are repeated 20 times to verify the reliability of the estimations.

As illustrated in the Response figure 7, the Regret model partially explain the observed empirical data. The accumulated impacts of regrets, as expected, is the part that allowed decision thresholds to have shallower slopes than the Optimal. Note that the current Regret model assumed perfect memory, which simulates the maximum effect of previous stimuli value, and thus, was provided with the maximal power to explain “flat” decision thresholds across opportunities induced by the regret (see Response figure 8 for the model that assumes limited memory capacity). Our simulation results suggest that, although we cannot rule out the impact of regret, the regret is not the major factor that underlies the behavioral suboptimality.

Because the model is a combination between the Optimal decision model and the regret, decision thresholds at the initial opportunities are always the same as those from the Optimal decision model. That said, we acknowledge and would like to clarify that the distinctive deviation at the initial opportunities in Experiment 3 between the Regret model and empirical data should not be used as the evidence against the potential impact of selection bias, and thus added an explicit description about its interpretation for readers.

**Response figure 7 (now added as figure S13A in SI).** Decision thresholds simulated with a regret model are compared against the optimal decision model and empirical data.

**Response figure 8.** Decision thresholds simulated with a variant of the regret model where only the most recent past stimulus is considered. As expected, restricted capacity of memory of the past stimuli minimizes the deviation the model can explain (i.e., remaining similar to the Optimal decision model).

To quantitatively assess the impacts of past stimuli values on the subsequent choice, we ran the same logistic regression analyses as we reported in **figure S9**. The regret model predicts meaningful deviation from the optimal model in the 3^rd^ and 4^th^ opportunities, and the deviation can be attributed to the negative weights on the preceding opportunities. However, the Regret model, inconsistent with the observed pattern from the empirical data (**Fig. S9**), predicts that the stimuli values from the earlier opportunities would affect the current decision to the greater extent compared with the stimuli values from the recent past. This subtle difference again suggest that the regret might not be the major factor underlying the observed behavioral suboptimality.

Of note, due to the deterministic nature of our simulation maximizing the impact of past stimuli values, we simulated larger number of choice rounds rather than using the same number of rounds as in the actual experiment (200 rounds), so that we can get a stable estimation of regression coefficients. Downsizing the simulation to 200 rounds as well as assuming the impact of only the most recent past stimulus value (current-1) still showed consistent results.

**Response figure 9 (now added as figure S13B in SI).** Effects of past stimuli values on the subsequent choice about the current value within the simulated data from a regret model.

To deepen our discussion, we included the description and results from the Regret model as one of the alternative models as following.

[Page 3, Supporting Information]

Besides the examined utility models, we further explored alternative mechanisms that could be recruited and deviate individuals’ decision patterns away from the optimal decision thresholds. Here, we explain these alternatives with simulation results.

**Regret model.** It is possible that individuals may regret about the past choices, and thus less likely to accept the stimuli values they previously rejected. To more directly examine potential effects of “regret”, we simulated behavioral choices with additional computational model where participants would accept candidates that are higher than both the optimal threshold and the highest value they have passed up in previous opportunities. To examine the maximum impact of past stimuli values, we applied the aforementioned rule of regret as deterministic decision thresholds at each opportunity and also assumed perfect memory (i.e., all the past stimuli values, rather than the most recent stimulus, affect decisions within the corresponding round). We simulated 10000 rounds of behavioral choices of pseudo-subjects, and estimated the decision thresholds and the effects of past values on the current decision. These steps are repeated 20 times to verify the reliability of the estimations.

The Regret model partially explain the observed empirical data (**Fig. S13A**). The accumulated impacts of regrets, as expected, is the part that allowed decision thresholds to have shallower slopes than the Optimal. Note that the current Regret model assumed perfect memory, which simulates maximum effect of previous stimuli value, and thus, was provided with the maximal power to explain “flat” decision thresholds across opportunities induced by the regret. Our simulation results suggest that, although we cannot rule out the impact of regret, the regret is not the major factor that underlies the behavioral suboptimality.

Because the model is a combination between the Optimal decision model and the regret, decision thresholds at the initial opportunities are always the same as those from the Optimal decision model. Note that in this simulation-based analysis, we are using the differences in slopes across decision thresholds, rather than other features (e.g., the deviation at the initial opportunities in Experiment 3), as criteria to examine whether the simulation model captures the core mechanism explaining the empirical data.

[Figure S13] Subjective optimality model versus an alternative Regret model

As examined in figure S9, there are potential effects of past stimuli values on the subsequent choice. (A) To more directly examine potential effects of “regret”, individuals accept less likely the stimuli values that are above the optimal threshold but below a value which they have previously rejected, decision thresholds simulated with a regret model are compared against the optimal decision model and empirical data. Error bars indicate s.e.m. (B) Effects of past stimuli values on the current decision were examined by applying the logistic regression analyses using the simulated data from the regret model. The regret model predicts meaningful deviation from the optimal model in the 3^rd^ and 4^th^ opportunities, and the deviation can be attributed to the negative weight on the preceding opportunities. However, the regret model, inconsistent with the observed pattern from the empirical data (Fig. S9), predicts that the value in the earlier opportunities will affect the current decision more than the value in the recent past. Error bars indicate s.e.m.

Besides the aforementioned edits, we fully agree with the points both the reviewer and the editors raised about insufficient evidence for the Subjective optimality model over the linear model [1], and thus, further analyzed behavioral data to highlight a unique behavioral feature that only can be explained by our suggested model. Specifically, decision thresholds calculated from empirical data showed a decreasing pattern along the opportunities (Fig. 1b, 3a, 4b), but moreover, the extent of threshold change between opportunities also showed a decreasing pattern (i.e., second derivative of the decision threshold curve < 0). Such a qualitative difference between the linear threshold model and the empirical data was corroborated by the observed statistical difference across opportunities in all four experiments (1^st^-2^nd^ vs 2^nd^-3^rd^ vs 3^rd^-4^th^; Exp1: F(2, 38) = 14.13, η^2^ = 0.43, p = 2.59e-05; Exp2 (K=5): F(2, 40) = 20.10, η^2^ = 0.50, p = 9.06e-07; Exp2 (K=10): F(7, 119) = 3.46, η^2^ = 0.17, p = 0.0021; Exp3: F(2, 40) = 25.01, η^2^ = 0.56, p = 9.00e-08). As illustrated below in Response figure 3, such a change in the slope of decision threshold curve is a unique feature that our suggested Subjective optimality model successfully could explain (differences between the model and empirical data do not vary across opportunities; Exp1: F(2, 38) = 0.70, η^2^ = 0.036, p = 0.090; Exp2 (K=5): F(2, 40) = 2.37, η^2^ = 0.11, p = 0.11; Exp2 (K=10): F(7, 119) = 0.67, η^2^ = 0.039, p = 0.70; Exp3: F(2, 40) = 1.80, η^2^ = 0.082, p = 0.18), and has superior explanatory power to the linear threshold model.

**Response figure 10 (now added as figure S11 in SI).** The slope of decision threshold curve decreases along the opportunities. Negative values on the y-axis indicate that decision thresholds decrease between opportunities.

[Page 21, Line 472]

The conclusions of the present study regarding the subjective valuation recruited in determining decision thresholds differ from previous findings by Baumann et al. [1], who argued that people rely on a mental heuristic and determine their thresholds linearly. As illustrated in our formal model comparison (**Fig. S1**; **Supplementary text**), individuals’ behavioral choices in each opportunity were explained by the Linear threshold model as well as by the Subjective optimality model. However, the decision patterns between opportunities were not accounted for by a linear decrease in decision thresholds, which is the main feature of the Linear threshold model (**Fig. S11**; **Supplementary text**). This discrepancy suggests that individuals’ choice data at least in the current variant of Secretary problem tasks cannot be fully explained by linear heuristic processes. Nevertheless, subtle differences in task designs (e.g., stimuli values followed a normal distribution in Baumann et al. [1], but a uniform distribution in the current study) may recruit different cognitive strategies, and thus future studies should explore potential factors that affect individuals’ decision processes (see **Supplementary text** and **Fig. S11-13** for further discussions).

**2. Regarding my second comment on the motivation for assuming prospect theory evaluation/a reference point, I take the authors’ point that empirically a fixed reference point in their model provides the best fit to the data. However, as I try to understand how the prospect theory pieces in the model account for behavior, I wonder whether the reference point is playing a major role or not. On page 10, the authors say that the subjective optimality model reduces to the optimal model if the value sensitivity parameter equals one (ie, if utility is a linear function of value). This suggests that the reference point is irrelevant if utility is linear. Could a model that assumes a non-linear utility function but has no reference point (expected utility) account for the data? What about a model that assumes a reference point plus loss aversion (linear utility with different slopes for gains and losses)? The broader point is about the motivation for the current model specification – the authors assume some pieces of prospect theory, but not others, and how those specific pieces are motivated by the data is not entirely clear.**

**Response:** We regret that we did not fully explore other pieces of prospect theory (e.g., loss aversion) and that we omitted the justification of our approach. Below, we provide the new results from the two additional models the reviewer suggested and specified about the edits we made in our manuscript for clarification.

As the reviewer pointed out, the Prospect Theory (PT) model is a general form of the other models including Expected Utility Theory (EUT) and Expected Value Theory (EVT). Note the model parameter estimation results that the reference point which best explains empirical data was around 100 and the value sensitivity was around 0.6. These parameter estimation results suggest that non-linear utility functions and non-zero reference points better explain participants’ choice behaviors.

Nevertheless, for completeness, we tested two additional models where 1) one assumes a non-linear utility function without a reference point (EUT) and 2) one assumes a linear utility function, a reference point, and a loss aversion. Across all experiments, both the EUT (labeled as ExpUtil) and the linear loss aversion (labeled as LinearLA) models showed inferior model fits (ExpUtil: 15139, LinearLA: 15288) compared to the Subjective optimality model with a waiting cost (SubjOpt: 15077). In each experiment, on the other hand, these model family (i.e., PT, LinearLA, and ExpUtil) showed comparable model-fit, equally better than the Optimal decision model (Response figure 11).

**Response figure 11 (now added as figure S1 in SI).** Model comparisons using Akaike Information Criterion (AIC)

Further assessment of each model’s estimated parameters revealed rather subtle but crucial lack of plausibility of both the LinearLA and the ExpUtil models. First, in the LinearLA model, mean of the loss aversion parameter was 0.23±0.03 (reference point = 98.77±15.63), indicating extremely strong *loss* *seeking* in individuals. This result is inconsistent with the rich previous literature on *loss* *aversion* that typical healthy individuals are reported to show (larger than 1). Second, in the ExpUtil model, mean of the value sensitivity (often referred to as ‘risk preference’) was 2.88±1.04, indicating extremely strong *risk seeking* in individuals. This result also is inconsistent with previous reports where typical healthy individuals are reported to have *risk aversion* (smaller than 1) in a gain frame (decision about positive rewards).

Based on these implausible parameters, we can conclude that it is unlikely for participants to use these values functions in their valuation and decision-making processes. Still, it seems worthwhile to discuss how come the models had comparable model-fit (besides the fact that the Subjective optimality model has one additional parameter (for the ExpUtil model), so that the model was penalized further in model comparison). The atypical parameters in the two alternative models (i.e., LinearLA, ExpUtil) correspond to a convex utility function across the full range of stimuli values. Note that the shape of these value functions provide similar coverage with that of our suggested model (see Response figure 12), which includes a high reference point (here, 110.51; see **Fig. 2B**) in combination with a concave value function for the gains (value above the reference point) and a convex value function for the losses (value below the reference point). This similarity between the shapes of value functions suggest that a broader range of stimuli values would be necessary to better dissociate each model’s explanatory power.

**Response figure 12.** Estimated utility functions for the Subjective optimality, Linear loss aversion, and Expected utility models.

Still with the limitation, we would like to emphasize once more about the strength of our suggested model. Even with its higher complexity (PT instead of EUT), inclusion of the reference point provided the model a superior generalization capacity across different contexts (e.g., different prior knowledge about maximum, different number of opportunities). We believe such a strength exceeds the aforementioned limitation. Below, we duplicated the edits and additions we made in the Supporting Information to clearly state the limitation, strength, and novelty of the current study with model descriptions.

[Page 2, Supporting Information]

Subjective utility function model justification

Based on the framework of Prospect theory [5], our suggested computational model introduced a subjective value function that includes a reference point and a nonlinear value sensitivity. Note that in our suggested model, we assumed that probability information is not distorted (linear probability weighting function) and that individuals are equally sensitive to gains and losses (loss neutrality). By doing so, we sought for simplicity while examining the impact of given contexts with a reference point, the key premise of Prospect theory [6]. Here, we tested two other models those belong to the same model family to further explore the impacts of simplified elements.

**Expected utility model.** As one of the special cases of the Subjective optimality model, we examined the effect of a non-linear utility function without a reference point. Across all experiments, the Expected utility model (labeled as ExpUtil) showed inferior model fit (ExpUtil: 15139) compared to the Subjective optimality model with a waiting cost (SubjOpt: 15077). In each experiment, on the other hand, these model family (i.e., PT and ExpUtil) showed comparable model fits, equally better than the Optimal decision model (**Fig. S1**). These results suggest that inclusion of a non-linear utility function has an important role in explaining the empirical data.

Further assessment of the estimated parameters included in the ExpUtil revealed rather subtle but crucial lack of plausibility. In the ExpUtil model, mean of the value sensitivity (often referred to as ‘risk preference’) was 2.88±1.04, indicating extremely strong *risk seeking* in individuals. This result is inconsistent with previous reports where typical healthy individuals are reported to have *risk aversion* (smaller than 1) in a gain frame (decision about positive rewards).

**Linear loss aversion model.** Another main component suggested in Prospect Theory other than a reference point is the enhanced sensitivity to losses relative to gains. To directly examine the impact of this ‘loss aversion’, we tested a linear utility function with inclusion of a reference point and a loss aversion. Across all experiments, the linear loss aversion (labeled as LinearLA) model showed inferior model fit (LinearLA: 15288) compared to the Subjective optimality model with a waiting cost (SubjOpt: 15077). In each experiment, on the other hand, these model family (i.e., PT and LinearLA) showed comparable model fits, equally better than the Optimal decision model (**Fig. S1**).

Further assessment of the estimated parameters included in the LinearLA revealed rather subtle but crucial lack of plausibility. In the LinearLA model, mean of the loss aversion parameter was 0.23±0.03 (reference point = 98.77±15.63), indicating extremely strong *loss* *seeking* in individuals. This result is inconsistent with the rich previous literature on *loss* *aversion* that typical healthy individuals are reported to show (larger than 1).

**Discussion about non-linear utility function family.** Based on the implausible parameters estimated for the ExpUtil and LinearLA models, we can conclude that it is unlikely for participants to use these values functions in their valuation and decision-making processes. Still, it seems worthwhile to discuss how come the models had comparable model-fit (besides the fact that the Subjective optimality model has one additional parameter (for the ExpUtil model), so that the model was penalized further in model comparison). The atypical parameters in the two alternative models (i.e., LinearLA, ExpUtil) correspond to a convex utility function across the full range of stimuli values. Note that the shape of these value functions provide similar coverage with that of our suggested model, which includes a high reference point (here, 110.51; see **Fig. 2B**) in combination with a concave value function for the gains (value above the reference point) and a convex value function for the losses (value below the reference point). This similarity between the shapes of value functions suggest that a broader range of stimuli values would be necessary to better dissociate each model’s explanatory power. Nevertheless, inclusion of the reference point in the Subjective optimality model provided the model a superior generalization capacity across different contexts (e.g., different prior knowledge about maximum, different number of opportunities). We believe such a strength exceeds the aforementioned limitation.

**3. Regarding my fourth comment on waiting cost, I take the authors’ point that changes in waiting cost across experiments fit the data. But the waiting cost is doing significant work in accounting for the major differences across experiments (whether thresholds are above the optimal in Experiments 1 and 2 or below in Experiment 3) and within experiments (in accounting for differences between K=5 and K=10 in Experiment 2). Yet waiting cost is not a part of the model that seems to be of theoretical importance in the paper. Why would waiting cost go up with more opportunities (K=10 in Experiment 2)? Is it really the delayed response requirement in Experiment 3 that increases waiting cost? Maybe the requirements of pupillometry (keeping head still, for example) make the subjects less tolerant of waiting? The current data can’t show for certain what is causing the differences in waiting cost. The paper is framed broadly in terms of the differences between an optimal model (expected value) and subjective optimality (prospect theory), but then the waiting cost parameter is doing much of the work.**

**Response:** We agree with the reviewer’s assessment about waiting cost having an important role, but would like to argue that waiting cost *does* have theoretical importance. Various theoretical and experimental studies took into account physical and/or mental efforts as a cost (or cost function) in subjective valuation [for review, see 7, 8]. In line with the literature, we assumed that individuals would take into account a mental cost (referred to as ‘waiting cost’) in valuation processes. Such an assumption is also linked with previous other studies where a version of secretary problem is used with their focuses being on the impact of sampling cost [9-11]. That said, note that the ‘waiting cost’ parameter introduced in our model simply is a linear disutility (negative subjective value), which captures a broad range of mental costs including not only the discomforts participants might have experienced during Experiment 3, but also the expected levels of mental efforts needed for sampling. As the reviewer noticed, K=10 in Experiment 2 is added in the process of the first round of revision, and therefore, we did not have any specific hypothesis stating that the waiting cost will capture the changes in the number of opportunities. Still, the waiting cost parameter estimated from the empirical data is consistent with our interpretation regarding mental cost, and thus showcasing generalizability of our suggested computational model.

We agree with the reviewer’s comment that the current data not being able to pin point what is causing the differences in waiting cost. However, as the reviewer noticed, the source of the waiting cost is not the focus of the current work. In the current revision, we edited the discussion section to clarify our interpretation about the parameter, and further added details about the optimal model that includes a waiting cost parameter (Opt+wc in Fig. S1) in the Supporting Information to highlight that it is *not* the waiting cost parameter that is crucial for explaining empirical data. The edits we made is attached below.

[Page 20, Line 432]

Our model parameter estimates support this interpretation, such that the estimated cost was significantly larger than zero in Experiment 3 where individuals were provided with additional forced waits and in Experiment 2 with more abundant opportunities (K = 10) where individuals were given with further opportunities.

[Figure S1] Note that a simple addition of the waiting cost does not improve the explanatory power of the Optimal decision threshold model, suggesting that the shape of subjective utility function has more crucial role.

**4. Regarding my fifth comment on the pupil data, I thank the authors for clarifying the multiple comparisons correction for the association between pupil diameter and value sensitivity. It is still not clear to me why we would expect this relationship, though, and it is really the only link between the pupil data and the model/rest of the paper. The findings of pupil associations with choice and choice difficulty are (nice) replications of previous work, but they don’t hinge on the specifics of the subjective optimality model.**

**Response:** Thank you for this comment. As noted in our manuscript, we are making the link between our suggested computational model with the pattern of pupil dilation data based on the hypothesis that neurophysiological responses would reflect individual-level characteristics in subjective valuation. Such model-based analysis approaches are very frequently used in neuroimaging studies where one searches for *neurobiological instantiations* of a certain information processing suggested by a computational model [12-14]. The specific association we hypothesized here is that, given the well-known relationship between pupil dilation responses and choice (and decision difficulty), individuals who have higher value sensitivity in their behavioral choices would also show higher (more sensitive) neurophysiological responses in pupil measures. We report that individual differences measured in behavioral and pupil measures are consistent one another, and use this behavior-to-pupil association as supporting evidence for our suggested valuation model.

**Reviewer #3: This is a very thorough and comprehensive revision, and I appreciate the authors' effort. All of my comments have been addressed.**

**Response:** Thank you very much again for taking time to review our work.

**References**

1.	Baumann C, Singmann H, Gershman SJ, von Helversen B. A linear threshold model for optimal stopping behavior. Proceedings of the National Academy of Sciences. 2020;117(23):12750-5.

2.	Tversky A, Kahneman D. Judgment under uncertainty: Heuristics and biases. science. 1974;185(4157):1124-31.

3.	Diederich A, Trueblood JS. A dynamic dual process model of risky decision making. Psychological review. 2018;125(2):270.

4.	Diehl K, Poynor C. Great expectations?! Assortment size, expectations, and satisfaction. Journal of Marketing Research. 2010;47(2):312-22.

5.	Kahneman D, Tversky A. Prospect theory: An analysis of decision under risk. Econometrica: Journal of the Econometric Society. 1979:263-91.

6.	Bendor J. Bounded rationality. 2001.

7.	Kool W, Botvinick M. Mental labour. Nature human behaviour. 2018;2(12):899-908.

8.	Inzlicht M, Shenhav A, Olivola CY. The effort paradox: Effort is both costly and valued. Trends in cognitive sciences. 2018;22(4):337-49.

9.	Costa VD, Averbeck BB. Frontal–parietal and limbic-striatal activity underlies information sampling in the best choice problem. Cerebral cortex. 2015;25(4):972-82.

10.	Lorenzen TJ. Optimal stopping with sampling cost: the secretary problem. The Annals of Probability. 1981;9(1):167-72.

11.	Seale DA, Rapoport A. Sequential decision making with relative ranks: An experimental investigation of the" secretary problem". Organizational behavior and human decision processes. 1997;69(3):221-36.

12.	Chung D, Christopoulos GI, King-Casas B, Ball SB, Chiu PH. Social signals of safety and risk confer utility and have asymmetric effects on observers' choices. Nature neuroscience. 2015;18(6):912-6.

13.	Tom SM, Fox CR, Trepel C, Poldrack RA. The neural basis of loss aversion in decision-making under risk. Science. 2007;315(5811):515-8.

14.	Kable JW, Glimcher PW. The neural correlates of subjective value during intertemporal choice. Nature neuroscience. 2007;10(12):1625-33.
