# Supplementary material for: Subjective optimality in finite sequential decision-making
Source: PLoS Comput Biol. 2021 Dec 16;17(12):e1009633. doi: 10.1371/journal.pcbi.1009633 (PMC8675647; doi:10.1371/journal.pcbi.1009633)
Supplement: Attachment — Submitted filename: Response_to_reviewers-R3.docx [file pcbi.1009633.s004.docx]

Dear Dr. Gershman and Dr. Haith,

Thank you very much for considering our manuscript PCOMPBIOL-D-20-01425R2 (“Subjective optimality in finite sequential decision-making”) for publication in PLOS Compuational Biology.

We have revised the manuscript and supporting information according to the indicated guidelines. These revisions are listed below:

- revised parts of the main text to address the results distinguishing the Prospect-theory-based model from alternative models
- edited figures and methods section to include essential results and methods for the additional analyses
- edited discussion section to clarify our approach and to note the limitation in interpreting the results regarding a waiting cost and pupillometry correlation

The reviewers’ comments are fully quoted below in bold; our responses follow each comment. Changes to the submission in response to the  reviewers’ comments are highlighted in blue in the revised manuscript and supplementary materials and also reproduced or summarized below.

**--**

**Dear Dr Chung,**

**Thank you very much for submitting your manuscript "Subjective optimality in finite sequential decision-making" for consideration at PLOS Computational Biology. As with all papers reviewed by the journal, your manuscript was reviewed by members of the editorial board and by several independent reviewers.**

**The reviewers have not reached a consensus on this paper. However, we feel that there is value in this work. The majority of the issues raised by the reviewers have been adequately addressed, and new analyses provide evidence that the prospect-theory-based model can account for aspects of behavior not captured by existing models.**

**However, as the reviewers noted, although the responses to the reviewers concerns were very thorough, many of these issues are still not addressed in the main text of the paper, despite being of critical importance in clearly establishing the contribution of the current paper. In particular, the comparison between the prospect-theory-based model and alternative models (e.g. the linear threshold model of Baumann et al.) is critical evidence supporting the new model (and, indeed, is critical to our editorial decision on this paper). However, serious consideration of these alternative models is raised only in the Discussion as something of an afterthought, rather than as a key component of the argument in favor of the prospect-theory model.**

**Given the strengths of the work, we would be willing to accept the paper, pending some minor revisions. We would like you to ask you to revise parts of the main paper to more prominently address some the issues that arose during the review process. Specifically, we feel it is critical to critical to distinguish the prospect-theory-based model from alternative models as a part of the Results, rather than just including these in the Supplementary Information (Currently Figures S11-13). It would also further strengthen the paper to use the Discussion to clearly articulate other limitations raised during review (e.g. those pertaining to the wait cost, expected findings from pupillometry, etc.).**

**Please prepare and submit your revised manuscript within 30 days. If you anticipate any delay, please let us know the expected resubmission date by replying to this email.**

**When you are ready to resubmit, please upload the following:**

**[1] A letter containing a detailed list of your responses to all review comments, and a description of the changes you have made in the manuscript. Please note while forming your response, if your article is accepted, you may have the opportunity to make the peer review history publicly available. The record will include editor decision letters (with reviews) and your responses to reviewer comments. If eligible, we will contact you to opt in or out**

**[2] Two versions of the revised manuscript: one with either highlights or tracked changes denoting where the text has been changed; the other a clean version (uploaded as the manuscript file).**

**Important additional instructions are given below your reviewer comments.**

**Thank you again for your submission to our journal. We hope that our editorial process has been constructive so far, and we welcome your feedback at any time. Please don't hesitate to contact us if you have any questions or comments.**

**Sincerely,**

**Adrian M Haith**

**Associate Editor**

**PLOS Computational Biology**

**Samuel Gershman**

**Deputy Editor**

**PLOS Computational Biology**

***************************

**A link appears below if there are any accompanying review attachments. If you believe any reviews to be missing, please contact ploscompbiol@plos.org immediately:**

**Reviewer's Responses to Questions**

**Comments to the Authors:**

**Reviewer #1: The authors have done an admirable job replying to my comments. I think it is clear that there is more going on here than just selection bias, regret, or linearly decreasing thresholds. I'm not totally convinced that the subjective optimality model is the singular best explanation, but it is grounded in solid theory (Prospect Theory) and seems to do better than any of the other proposed mechanisms on their own. In any case, the phenomenon is interesting and this paper will hopefully spur more work on this topic.**

**I do have one lingering concern that the authors should perhaps address in a minor revision. It has to do with the selection bias story. The authors treat the selection bias as a "model" and relegate it to the depths of the supplementary material (Figure S12) without any mention of it in the main text. I think this is a mistake. Selection bias is not a "model", it is a statistical phenomenon that is undoubtedly present in the authors' data. It is important to acknowledge and discuss it in the main text. Otherwise readers might overestimate the size of the deviation from "optimality" and future researchers might overlook this important detail of modeling such data. Personally, I would have liked to have seen a reasonable amount of selection bias incorporated into the other models (regret, linear threshold) and examined relative to the subjective optimality model in e.g. Fig. S11. However, I won't insist on this given where we are in the review process. But I would like to see more acknowledgment of this important statistical issue.**

**I also take issue with the results presented in Response Figure 2 (Fig. 12B) where the authors argue that the correlation between response variability and threshold change is considerably different in the data compared to the selection bias "model". This is obviously because of the assumptions in the authors' selection bias model. Specifically, the authors assume that that there is lots of across-round variability but no across-opportunities variability. In reality, there is surely both across-round and across-opportunity variability that are highly correlated. If that correlation was incorporated into the selection bias model, my guess is that it would more closely match the data. I would suggest that the authors either verify this, or simply omit Figure S12B from the paper. As it is now, I think that it is just misleading.**

**Response:** Thank you very much again for taking time to review our work, and we appreciate the reviewer’s positive assessments of our work.

Per the reviewer’s suggestion, we moved the section related to selection bias as well as the figure S12 to the main text (and as Fig. 8), and rephrased our description about the phenomenon to acknowledge its statistical nature.

[Page 19, Line 410]

**Alternative mechanisms and factors**

In the current study, we tested our hypothesis that individuals use subjective valuation and dynamic programming approach during finite sequential decision-making. Here, we further explored potential factors (e.g., regret, selection bias) that could be recruited and contribute to biasing behavioral choices.

[Page 20, Line 430]

**Selection biases.** It is possible that due to decision variabilities, participants’ choice rounds where they happened to have lower decision thresholds would be accepted at earlier opportunities, and thus be under-represented at subsequent opportunities. We ran a new simulation to implement how the behavioral pattern would become if the selection bias indeed is the major factor that biases individuals away from the optimal behavioral pattern (see **Methods** for simulation details). To test whether selection bias explains the flatness of the empirical decision thresholds, we first, as the most conservative setting, assumed that the entire behavioral choice variability contributes to the within-subject variability across rounds (see **Methods** for justification). Based on the simulated choices, the model that explicitly incorporated selection bias (‘Selection bias model’ hereafter) partially explained the deviation from the optimal thresholds and show relatively shallower slopes as expected (**Fig. 7A**). However, compared to empirical decision thresholds across opportunities, the simulation slopes were relatively steeper even though the simulation was run with the maximum level of variability as we described above. Note that in this simulation-based analysis, we are using the differences in slopes across decision thresholds, rather than other features (e.g., the deviation at the initial opportunities in Experiment 3), as criteria to examine whether the simulation model captures the core mechanism explaining the empirical data.

Although our simulation using the maximum level of within-subject variability across rounds was our attempt to give the Selection bias model the maximal power to explain “flat” decision thresholds across opportunities, we additionally examined the impact of different levels of within-subject variability on decision thresholds. There was a stark difference between the simulated and empirical data when probed through the association between within-subject variability across rounds and decision thresholds. Consistent with our description above, the largest response variability in the Selection bias model was associated with the smallest threshold change across opportunities, and vice versa (r = -0.97, *P* = 1.70e-40; **Fig. 7B**). It was also the case when part of the variability was attributed to across-opportunity rather than across-round variability in the simulation (**Fig. 7C**). On the contrary, empirical data revealed that participants who had the largest response variability showed the largest threshold change across opportunities (r = 0.43, *P* = 5.21e-04), which is the exact opposite of the Selection bias model. Together, these results suggest that selection bias cannot explain empirical decision patterns observed in this task.

Regarding the reviewer’s comment about the across-round variability, we ran additional set of simulations where not only across-round variability, but also across-opportunity variability was incorporated to explicitly test its potential impact on decision thresholds. Specifically, as depicted in the figure below (**Response figure 1**), we ran 11 separate simulations when part of the variability was attributed to across-opportunity and the rest attributed to across-round variability. In contrast to the reviewer’s expectation, as the proportion of variability attributed to across-opportunity variability increased, the threshold-change gradually increased (i.e., steeper curve) and converged to the level of Optimal decision model. The correlation between individuals’ response variability and threshold changes also varied as a function of the proportion of across-round variability. Specifically, if all of each individual’s response variability is attributed to across-round variability, as we reported in the previous revision, the correlation asymptotes to -1, whereas if all of the response variability is attributed to across-opportunity variability, the correlation becomes 0. This new set of results shows that across-round variability, rather than across-opportunity, is responsible for the decreasing threshold-change, and that the positive correlation observed from the empirical data cannot be explained by selection bias nor the mixture between across- round and opportunity variability.

**Response figure 1 (now added as Figure 7C).** To examine the impact of across- round versus opportunity variability, we ran additional simulations with varying proportion of across-round variability out of the maximum amount of variability. As the proportion of variability attributed to across-round against the across-opportunity variability, the threshold-change gradually decreased from the level of the optimal behavior, and the correlation between threshold-change and response variability asymptotes to -1, decreasing from 0. Levels of threshold change for the empirical data (red) and the Optimal model (blue) are illustrated together as references.

**Reviewer #2: I appreciate the new analyses the authors have added in response to my comments, but I am afraid we’re at an impasse. While I feel that the issues raised over the last two rounds of review should have merited re-framing the paper, or tempering its conclusions substantially, the authors disagree. The main body of the paper has not really changed in this revision; additions have been made to the supplement but in many cases these are not even mentioned in the main text.**

**1. Regarding my previous comment that there was an effect of previous candidate values which were passed over on current choices, the authors present a model that adds to the optimal a feature that people do not accept candidates that are above threshold but below what they’ve previously passed up. This model (with no free parameters) also has the feature that thresholds do not decline as steeply as in the optimal model, but does not account for the data as well as the authors’ model (which has four free parameters). While this suggests that a regret is not the only feature necessary to account for the data, it does not show that regret is having no effect on people’s choices at all. The significant effects of past values on current choices is not accounted for by the subjective optimality model. But this weakness in the model is not acknowledged.**

**Similarly, the data the authors present to rule out the linear threshold reduction model is simply a re-plotting of the data shown in the main figure. The subjective optimality model traces the data better than the linear threshold reduction model (though mostly in the change between the third to last and second to last opportunity). But the subjective optimality model also has more parameters. When penalizing for this complexity, there is no difference between the two models.**

**Response:** We regret that our previous revisions did not incorporate the alternative accounts. In the new revision, we moved the description and results about the Regret model to the main text, and edited to acknowledge potential alternative mechanisms including regret.

[Page 19, Line 410]

**Alternative mechanisms and factors**

In the current study, we tested our hypothesis that individuals use subjective valuation and dynamic programming approach during finite sequential decision-making. Here, we further explored potential factors (e.g., regret, selection bias) that could be recruited and contribute to biasing behavioral choices.

**Regret model.** It is possible that individuals may regret about the past choices, and thus less likely to accept the stimuli values they previously rejected. To more directly examine potential effects of “regret”, we simulated behavioral choices with additional computational model assuming that participants would accept the candidates those are higher than the highest value they have rejected before (see **Methods** for simulation details). The Regret model partially explain the observed empirical data (**Fig. 6A**). The accumulated impacts of regrets, as expected, is the part that allowed decision thresholds to have shallower slopes than the Optimal. Note that the current Regret model assumed perfect memory, which simulates maximum effect of previous stimuli value, and thus, was provided with the maximal power to explain “flat” decision thresholds across opportunities induced by the regret. Our simulation results suggest that, although we cannot rule out the impact of regret (see **Discussion** for further discussion about potential learning effects), the regret is not the major factor that underlies the behavioral suboptimality.

[Page 25, Line 556]

On the same line, we acknowledge that there might be other factors (e.g., regret, memory, selection bias) and their combination which would be able to explain choice patterns during the task, and thus future studies should explore potential factors that affect individuals’ decision processes.

[Page 27, Line 515]

Still, inclusion of learning mechanisms in the model (e.g., learning what to expect, regretting previous choices) would~

[Page 36, Line 798]

**Regret model.** We constructed a model where participants would accept candidates that are higher than both the optimal threshold and the highest value they have passed up in previous opportunities. To examine the maximum impact of past stimuli values, we applied the aforementioned rule of regret as deterministic decision thresholds at each opportunity and also assumed perfect memory. That is, all the past stimuli values, rather than the most recent stimulus, affect decisions within the corresponding round. We simulated 10000 rounds of behavioral choices of pseudo-subjects, and estimated the decision thresholds and the effects of past values on the current decision. These steps are repeated 20 times to verify the reliability of the estimations. Because the model is a combination between the Optimal decision model and the regret, decision thresholds at the initial opportunities are always the same as those from the Optimal decision model. Note that in this simulation-based analysis, we are using the differences in slopes across decision thresholds, rather than other features (e.g., the deviation at the initial opportunities in Experiment 3), as criteria to examine whether the simulation model captures the core mechanism explaining the empirical data.

Regarding the Linear threshold model, we would like to reiterate the value of the new figures we provided; the illustration highlights the feature, rather than simply describing overall model fit, that cannot be explained by the Linear threshold model, but by our suggested Subjective optimality model. In the current revision, following the editor’s and the reviewer’s suggestion, we moved the new results to main text from SI.

[Page 11, Line 232]

From the formal model comparison, we noticed that our suggested computational model showed comparable model-fit with some of the alternative models including the Linear threshold model, a model recently presented to well explain human stopping decisions in sequential decision-making [1]. Here, besides examining model fits, we examined whether the distinctive linear feature of the Linear threshold model presents in our empirical data. Decision thresholds calculated from empirical data showed a decreasing pattern along the opportunities (**Fig. 1b**), but moreover, the extent of threshold change between opportunities also showed a decreasing pattern (i.e., second derivative of the decision threshold curve < 0; **Fig. 2C**). Such a qualitative difference between the linear threshold model and the empirical data was corroborated by the observed statistical difference across opportunities ([1^st^-2^nd^] vs [2^nd^-3^rd^] vs [3^rd^-4^th^]; F(2, 38) = 14.13, η^2^ = 0.43, p = 2.59e-05). These results suggest that individuals’ choice data in the current variant of Secretary problem task cannot be fully explained by the Linear threshold model.

[Page 14, Line 303]

Besides the prediction approach, we also confirmed that empirical decision threshold curves from Experiment 2 also showed negative second derivative across contexts, and that Subjective optimality model captures the patterns (**Fig. 3D**).

[Page 16, Line 352]

In addition, as in both Experiments 1 and 2, empirical decision threshold curve showed negative second derivative, which is the feature that Linear threshold model, unlike our Subjective optimality model, cannot capture (**Fig. 4C**).

**2. Regarding my previous comment about the prospect theory specification (which has a reference point and non-linear utility), the authors present new analyses showing that other specifications (non-linear utility alone, or reference point plus linear utility plus loss aversion) do similarly. To my mind, what these fits reveal is that accounting for behavior requires adding to the optimal model a convex subjective transformation of the value space. The authors have reasons for favoring their transformation over others, but the specific convex transformation is not constrained by the data.**

**Response:** We fully agree with the reviewer’s comment regarding the shape of utility function. Our computational model is constructed based on Prospect theory and we adopted a standard power utility function to explain concave (or convex) utility across gains (losses). However, we are not arguing that the specific power utility function is the only utility model that explains the data.

**3. The authors did not make any changes in response to my previous comments about waiting cost. The waiting cost parameter still feels ad hoc to me (it’s not in Prospect Theory, for example, which is the inspiration for the model!), added to account for the differences between Experiment 3 and the others. But these results are discussed as if the differences in waiting cost were predicted by the model, or are a hypothesis confirmed by the model. To claim this as a prediction would require some hypothesis about what is driving the waiting cost that is directly tested.**

**Response:** We respectfully disagree with the reviewer’s comment regarding the waiting cost. Our suggested model is indeed drawn upon the Prospect theory. However, to explain individuals’ decision patterns during the secretary problem, a specific context different from the original setting of the original Kahneman & Tversky [2], we expanded the model so that our suggested model could include psychological construct of a waiting cost (as previous studies about Secretary problem reported its relevance) and capture all the hypotheses we set in the current study [3]. We acknowledge that our model cannot pinpoint where the waiting cost is originated from, but that is why we hypothesized it as a single component encompassing various types of mental cost and focused on its mechanistic involvement. To clarify our approach and provide a note about the limitation, we made the following changes in Introduction of the main text.

[Page 6, Line 113]

Various theoretical and experimental studies took into account physical and/or mental efforts as a cost (or cost function) in subjective valuation [for review, see 4, 5]. In line with the literature, we assumed that individuals would take into account a mental cost (referred to as ‘waiting cost’ hereafter) in valuation processes. Such an assumption is also linked with previous other studies where a version of secretary problem is used with their focuses being on the impact of sampling cost [6-8]. Thus, we also included a waiting cost [7, 8] parameter as a linear disutility (negative subjective value), which captures a broad range of mental costs during decision processes (see **Methods** for detailed model structure; see **Supplementary text** for detailed model justification).

[Page 23, Line 512]

In the current study, we hypothesized a waiting cost as a single component encompassing various types of mental cost and focused on its mechanistic involvement. Future studies exploring the source of the waiting cost may further our understandings about how and why individuals sometimes make choices impulsively or patiently.

**4. The authors also did not make any changes in response to the previous comments about the pupil data. It is still not clear to me why we would expect higher pupil diameter in people with greater value sensitivity, or how this prediction arises from the subjective optimality model.**

**Response:** The value sensitivity parameter is the parameter we introduced in our utility function that determines its concavity (or convexity). This parameter explains how objective values are subjectively perceived to each individual, and an individual’s small value sensitivity shows how fast one’s behavioral responses to presented values saturate. To reiterate our response in our previous revision, the association we hypothesized is between pupil dilation responses and individuals’ value sensitivity parameters because pupil dilation responses are known to reflect values (and other related dimensions including uncertainty, surprise, cognitive conflict, and choice). The correlation result we report indicates that ‘behavior-level sensitivity’ measure we extracted from our model is consistent with an independent ‘neurophysiology-level sensitivity’. Such consistency across data from different domains supports a mechanistic account of seemingly suboptimal decision strategies in finite sequential decisions. To clarify our interpretation, we made the following edits in Discussion section.

[Page 26, Line 586]

This correlation result indicates that ‘behavior-level sensitivity’ we extracted from our model is consistent with an independent ‘neurophysiology-level sensitivity’. Consistent with previous studies, our pupillometry results evidence the association between neurophysiological pupil responses and individuals’ characteristics in valuation [9], and support a mechanistic account of seemingly suboptimal decision strategies in finite sequential decisions.

**Reviewer #3: My original comments were addressed on the first revision, but the editors and other reviewers have made additional insightful questions, comments, and suggestions. Thus, my re-evaluation has focused on how the authors have addressed these issues.**

**Does the proposed model provide a better account of the data than existing models? In my view, the authors show that their model explains an aspect of behavior that is not accounted for by the linear threshold model. Specifically, they show that the slope of decision threshold decreases across opportunities. Although I don't fully understand why this relation is most pronounced in the last few opportunities, it seems like a clear advance over existing models.**

**I also think the authors were fairly thorough in their responses and revisions, but I defer to the other reviewers and editors about these specific issues since they were things that I did not personally raise. In any case, I appreciate the work that the authors and reviewers have put into this manuscript, and I think the product is much improved.**

**Response:** Thank you very much again for nicely summarizing the strength of our work and taking your time once more to review our revised manuscript.

**References**

1.	Baumann C, Singmann H, Gershman SJ, von Helversen B. A linear threshold model for optimal stopping behavior. Proceedings of the National Academy of Sciences. 2020;117(23):12750-5.

2.	Kahneman D, Tversky A. Prospect theory: An analysis of decision under risk. Econometrica: Journal of the Econometric Society. 1979:263-91.

3.	Wilson RC, Collins AG. Ten simple rules for the computational modeling of behavioral data. Elife. 2019;8:e49547.

4.	Kool W, Botvinick M. Mental labour. Nature human behaviour. 2018;2(12):899-908.

5.	Inzlicht M, Shenhav A, Olivola CY. The effort paradox: Effort is both costly and valued. Trends in cognitive sciences. 2018;22(4):337-49.

6.	Costa VD, Averbeck BB. Frontal–parietal and limbic-striatal activity underlies information sampling in the best choice problem. Cerebral cortex. 2015;25(4):972-82.

7.	Lorenzen TJ. Optimal stopping with sampling cost: the secretary problem. The Annals of Probability. 1981;9(1):167-72.

8.	Seale DA, Rapoport A. Sequential decision making with relative ranks: An experimental investigation of the" secretary problem". Organizational behavior and human decision processes. 1997;69(3):221-36.

9.	Eldar E, Felso V, Cohen JD, Niv Y. A pupillary index of susceptibility to decision biases. Nature Human Behaviour. 2021:1-10.
